# Novel Insights into the Mechanism and Treatment of Diabetes-Related Brain Complications: Focusing on the Blood-Brain Barrier Impairment

**DOI:** 10.14336/AD.2025.0296

**Published:** 2026-06-08

**Authors:** Caiyi Long, Yueheng Pu, Shunseng Keng, Jiajing Tao, Boxun Zhang, Rensong Yue

**Affiliations:** ^1^Hospital of Chengdu University of Traditional Chinese Medicine, Chengdu, China.; ^2^Chengdu University of Traditional Chinese Medicine, Chengdu, China.

**Keywords:** Blood-brain barrier, diabetes mellitus, cognitive impairment, stroke, depression

## Abstract

Diabetes mellitus often leads to secondary brain disorders, thus increasing the risk of mortality. The blood-brain barrier (BBB) is a peripheral-central defense mechanism that significantly impacts diabetes-related brain complications. Under hyperglycemic conditions, the BBB undergoes pathological structural alterations, leading to increased permeability and transport dysfunction. Clinically, BBB damage induces diabetes-related brain complications such as cognitive impairment, stroke, and depression. Notably, BBB damage can occur before the onset of disease symptoms in the brain and may serve as a predictor of disease progression and prognosis. Hyperglycemia is the main cause of BBB damage and can induce oxidative stress, inflammatory response, Advanced glycation end products (AGEs) accumulation, and high-mobility group box 1 (HMGB1) signaling axis activation. These factors lead to endothelial dysfunction, disruption of tight junction proteins, loss of pericytes, activation of astrocytes and microglia, disruption of the actin cytoskeleton, alterations in the basement membrane, and an increase in matrix metalloproteinases (MMPs). Collectively, these processes contribute to brain injury in patients with diabetes. Lifestyle interventions and hypoglycemic, antihypertensive, and lipid-lowering agents play therapeutic roles in BBB damage and diabetes-related brain complications. However, the role of some drugs in this context is controversial and remains known only at the animal and cellular levels. Several studies have investigated the therapeutic potential of targeted nanomedicines and natural compounds; however, it remains challenging to translate their research findings to clinical practice. In conclusion, this review highlights the clinical evidence, pathological mechanisms, and existing treatment options for BBB damage in patients with diabetes-related brain complications. It also demonstrates the potential of targeted nanomedicines and natural compounds, providing a foundation for future research.

## 1. Introduction

The high prevalence of diabetes mellitus (DM) poses a substantial ongoing global public health challenge, with type 2 diabetes mellitus (T2DM) accounting for approximately 90% of cases [1]. Hyperglycemia in patients with diabetes, along with insulin resistance, chronic inflammation, oxidative stress, genetic factors, and comorbidities (such as hypertension, dyslipidemia, and obesity), contribute to the development and progression of diabetes-related brain complications, particularly cognitive impairment, stroke, and depression, which have recently garnered significant attention [2-4]. A previous meta-analysis indicated that the risk of cognitive impairment, which also encompasses dementia, is 1.25 to 1.91 times greater for patients with DM [5]. Moreover, the prevalence of major depressive disorder (MDD) among patients with DM is reportedly as high as 46.6% [6]. With regard to ischemic stroke and hemorrhagic stroke, the prevalences are 33% and 26%, respectively, among patients with DM [7]; these conditions are twice as common as those in individuals without DM [8]. All these complications may coexist and exacerbate each other [2, 9, 10], not only burdening patients with higher healthcare costs [11] but also leading to a worse prognosis and a higher risk of all-cause mortality [12, 13].

The pathogenesis of these complications is complex and multifactorial. Research has shown that the blood-brain barrier (BBB), a key barrier between the central nervous system and peripheral tissues, also exhibits endocrine functions [14]. BBB damage induced by high glucose levels is closely associated with the development of cognitive impairment [15, 16], stroke [17], and depression [18, 19]. This damage may occur before visible disease symptoms manifest [20]; thus, it could be an early biomarker of diabetes-related brain complications and a crucial target for therapeutic interventions.

In this review, we explored the phenotypic and pathological mechanisms underlying BBB damage caused by diabetes. We review and discuss new evidence regarding BBB damage and its consequences on cognitive impairment, stroke, depression, and other diabetes-related brain complications. In addition, we have identified potential therapeutic interventions, providing valuable directions for future research.

## 2. Phenotypic alterations of the blood-brain barrier in diabetes: from physiology to pathology

BBB is a highly selective physiological barrier comprising specialized brain microvascular endothelial cells (BMECs) and their junctional complexes (e.g., tight junctions [TJs] and adherens junctions [AJs]), pericytes, astrocyte endfeet, basement membrane, and resting microglia [21-23].

Among these components, BMECs and their junctional complexes constitute a critical element of BBB, meticulously regulating molecular exchange between the blood and brain tissue. Barrier integrity depends on key TJ proteins such as claudin and occludin [24, 25], while zonula occludens proteins (primarily ZO-1 and ZO-2) serve as the principal scaffolding that anchors these transmembrane TJ proteins to the cytoskeleton [26, 27]. TJs control paracellular permeability. Claudin-5, the predominant TJ protein in BBB [28], permits the selective passage of molecules smaller than 800 Da while restricting larger molecules (> 800 Da) from diffusing via the paracellular route [29]. In addition to TJs, endothelial junctional complexes include AJs, which are primarily responsible for the establishment, maturation, and maintenance of endothelial cell-cell adhesion [30, 31].

Pericytes surround BMECs and collaborate with them to sustain the structural stability and functional integrity of BBB through direct intercellular interactions and signaling [32]. Astrocyte endfeet are closely associated with the vascular basement membrane, thus forming an almost continuous barrier that further enhances the protective function of BBB [33]. Furthermore, astrocytes release various growth factors and signaling molecules such as vascular endothelial growth factor (VEGF) and angiotensin-1. These factors play a significant role in regulating BBB permeability, supporting neuronal metabolism and maintaining homeostasis in the central nervous system (CNS) [34-36].

As inherent immune cells within CNS, microglia not only possess phagocytic abilities but also participate in the regulation of inflammatory responses and BBB permeability alterations through cytokine secretion and interactions with other cells [37, 38]. This complex network of cell-cell interactions contributes to efficient screening and precise regulation of blood components by BBB.

In terms of transport mechanisms, the BBB regulates the selective exchange of substances between the blood and brain tissue, primarily via passive diffusion, active efflux, carrier-mediated transport, receptor-mediated transport, and cellular trafficking across BBB. Passive diffusion of molecules across BBB primarily depends on their physicochemical attributes, e.g., molecular weight [39], electric charge [40], and high lipid solubility [41], which facilitate their transcellular passage through the tightly packed endothelial cells. Active efflux removes certain materials from the brain through ABC transporters, e.g., P-glycoproteins (P-GP), breast cancer resistance proteins (BCRPs), and multidrug resistance proteins (MRPs) [42-44]. Carrier-mediated transport sets up a selective influx-efflux system, while glucose transporter proteins and amino acid transporters play vital roles in providing nutritional support to the brain [45, 46]. Receptor-mediated transport identifies and binds macromolecules through specific receptors such as the transferrin receptor, low-density lipoprotein receptor-related protein 1 (LRP1), and the receptor for advanced glycosylation endproducts (RAGE), facilitating their transport across the cell membrane [47-49]. Cellular trafficking involves immune cells invading the brain through BBB to mediate both the central and peripheral immune responses [50]. These transport systems collectively establish a highly regulated transcellular pathway at BBB, facilitating the tight regulation of essential nutrients, metabolic substrates, and signaling molecules, among others, into and out of CNS.

**Figure 1.**
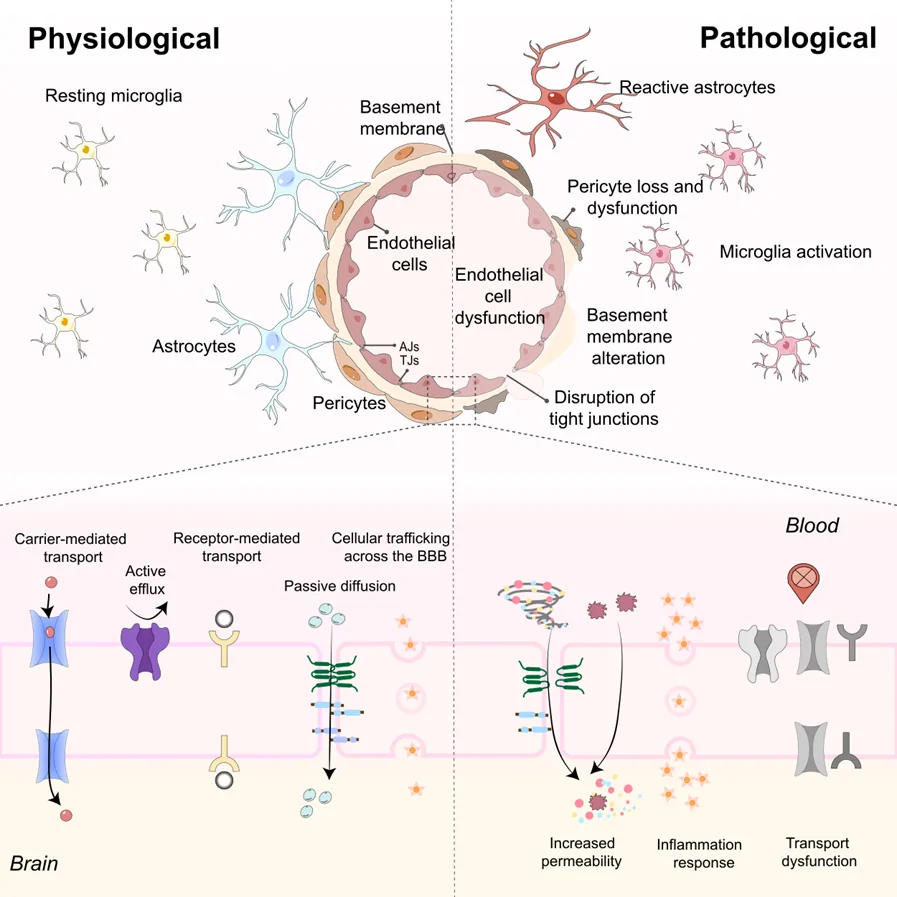
**Phenotypic changes in blood-brain barrier damage**. The left side of the figure shows the principal structural components and transport pathways of the blood-brain barrier under physiological conditions. The right side depicts the structural disruption and functional impairment of the blood-brain barrier under pathological conditions.

In the pathological state of diabetes, structural and functional impairments occur in vascular cells and glial cells of BBB; these include (a) disruption of endothelial cells and their TJs [51-58], (b) dysfunction and loss of pericytes [53, 54, 59, 60], (c) activation of microglia [59], (d) activation and crinkling of astrocytes [60], (e) damage to vascular structures [61-64], and (f) thickening of the basement membrane [65]. These morphological changes disrupt BBB integrity, leading to dysfunction characterized by increased permeability and transport deficiencies. In clinical studies, increased BBB permeability manifests as an increased cerebrospinal fluid (CSF)/serum albumin quotient (QAlb) [66-69], altered expression of proteins such as intercellular adhesion molecule-1 (ICAM-1), vascular cell adhesion molecule-1 (VCAM-1), and VEGF [68, 69], and altered brain imaging findings [70, 71]. In animal and cellular experiments, this is shown as loss of TJs; leakage of the immunoglobulin G (IgG) index [72]; enhanced permeability of albumin [64, 65], inulin [51], and [14C] sucrose [51]; and upregulation of fluorescein (FL) [73]. BBB transport dysfunction mainly involves abnormal levels of transport proteins, such as P-GP, BCRP, and glucose transporter type 1 and 4 (GLUT1 and GLUT4), as well as impaired cation transport. The main structures and functions are shown in Figure 1.

## 3. BBB damage affects diabetes-related brain complications: clinical evidence

### 3.1 Cognitive impairment

Patients with diabetes exhibit increased BBB permeability, and this increase may contribute to further clinical deterioration in individuals with cognitive impairment [66, 67]. Evidence indicates that these patients show increased BBB permeability compared with that in individuals without diabetes, as shown by elevated Qalb values and high levels of ICAM-1, VCAM-1, and VEGF [66-70, 74]. In imaging studies, patients with T2DM have also demonstrated increased gadolinium diethylenetriaminepentaacetic acid (Gd-DTPA) enhancement [71] and elevated K^trans^ values [75]. Cerebral blood flow (CBF) is similarly linked to BBB permeability; clinical data indicate that every 2.2-mL/min/100 g decrease in CBF corresponds to a 0.7% increase in BBB leakage [76]. In addition, research has focused on the association between cognitive impairment and altered cerebral hemodynamics in T2DM [77]. A case report indicated a link between cognitive impairment and cerebral microangiopathy in patients with acute diabetes [78], while a cohort study revealed a 42% increase in the risk of dementia in individuals with diabetic retinopathy [79].

BBB damage promotes the progression of diabetic cognitive impairment (DCI). A cohort study found that the risk of DCI progression increased by 8% with every 10% increase in log-Qalb levels [66]. Another 17-month cohort study demonstrated that elevated QAlb serves as a significant predictor of DCI progression [67]. An 8-12-year cohort study by Mielke et al. found that elderly patients with T2DM showing overweight or obesity had higher plasma neurofilament light chain (NfL) and glial fibrillary acidic protein (GFAP) levels, which are linked to later cognitive decline and a greater risk of cognitive impairment [80]. Therefore, it is crucial to recognize that impaired BBB permeability can precede cognitive decline. However, one study has also shown that increased BBB permeability does not correlate with cognitive function, likely because cognitive decline typically emerges as a late-stage biomarker, and the relationship between BBB permeability and cognitive decline may take time to develop [70].

### 3.2 Stroke

The incidence of stroke is higher for patients with DM than for individuals without diabetes, often leading to poor prognoses in the former [81-83]. BBB damage is a significant feature of early brain injury following stroke [84-86]. This is primarily indicated through brain imaging parenchymal enhancement (PE) signals, K^trans^, blood-retinal barrier (BRB) dysfunction, and relevant biocirculatory markers. A study examining BBB damage on the basis of PE in T1-weighted imaging with a 5-minute delay after contrast injection revealed that patients with diabetes showing varying PE increases experienced adverse stroke outcomes [87]. Kawamura et al. found that patients with T2DM who had silent cerebral infarcts showed higher levels of soluble ICAM-1 (sICAM-1), soluble E-selectin, and soluble VCAM-1 than did those without infarcts, and that patients with a baseline sICAM-1 level of >260 μg/L had a higher risk of stroke [88]. Moreover, an 8-year cohort study reported that patients with T2DM and cerebral infarction had significantly elevated sICAM-1 levels, and that sICAM-1 was an independent risk factor for stroke in these patients [89].

Another study showed a negative correlation between brain-derived neurotrophic factor and hyperglycemia, along with elevated endothelial progenitor cell counts, in post-stroke patients with diabetes [90]. BRB is functionally and structurally similar to BBB and may reflect BBB integrity. A 10-year clinical study indicated that patients with DM and BRB dysfunction tended to experience stroke more quickly [91].

BBB permeability assessment also helps predict the prognosis of patients with clinical stroke. Research has shown that evaluation of BBB permeability using perfusion computed tomography can predict the risk of symptomatic hemorrhagic transformation and malignant edema in patients with ischemic stroke [92].

### 3.3 Depression

DM and depression often co-occur because of shared underlying pathological mechanisms, e.g., inflammation, metabolic dysfunction, oxidative stress, and insulin resistance [93]. A meta-analysis indicated a 40% prevalence of comorbid depression in patients with DM [94]. Depression is associated with a nearly 1.5-fold increased risk of death in patients with DM [95], greatly impacting their quality of life.

Clinical findings have shown that patients with depression often have increased BBB permeability and transport dysfunction [96-98]. Biomarkers such as Qalb [96], S100B protein [99], sICAM-1 [100], VEGF [101], and proinflammatory cytokines such as interleukin (IL)-6 and tumor necrosis factor alpha (TNF-α) [102] were found to be elevated in patients with MDD. A human genetics study suggested a notable association between altered P-GP and MDD, indicating a potential relationship between BBB transport dysfunction and MDD progression [103]. Some studies have explored the relationship between diabetes, depression, and BBB function. Insulin resistance and related inflammatory pathways can lead to BBB leakage, thereby exacerbating depression [104, 105]. In patients with T1DM, higher serum sICAM-1 levels correlate with worse depressive symptoms [100]. In patients with T2DM, depressive symptoms measured using serial Patient Health Questionnaire-9 scores positively correlate with VEGF levels [101]. Similarly, a meta-analysis suggested that depression in patients with diabetes is associated with an increased risk of macrovascular and microvascular complications [106]. Another cross-sectional study found that retinal vascular calibers were significantly wider in patients with T2DM showing depression than in patients with diabetes without depression and healthy controls, suggesting early microvascular changes in patients with DM showing depression [107].

Diabetes-related brain complications often occur simultaneously or sequentially [108-110]. Diabetes is an independent predictor of poor cognitive recovery following stroke [111]. In addition, poorer glycemic control [109], long duration of diabetes [112], and high levels of glycosylated hemoglobin [112] are risk factors for cognitive impairment following stroke in patients with T2DM. Cognitive impairment is also associated with an increased risk of stroke in patients with diabetes [113]. Depression is an independent risk factor for cognitive impairment [114]. A study of 5,263 individuals showed that patients with diabetes showing depression had a subjective decline in cognitive abilities [110]. These findings suggest that BBB damage underlies diabetes-associated brain complications, forming a self-perpetuating loop that accelerates disease progression.

### 3.4 Other brain diseases

BBB damage in diabetic patients may also contribute to the development of other neurological disorders such as epilepsy [115] and tuberculous meningitis [116]. However, only preclinical animal studies have been reported on conditions such as Parkinson’s disease and anxiety [117, 118], and further clinical evidence is needed for confirmation.

A case report described a patient with nonketotic hyperglycemia (NKH) who exhibited intermittent jerky movements of the left arm and blurred vision in the left visual field of both eyes following intravenous gadolinium injection, which showed a tiny overlying area of interstitial CSF enhancement around the right parietal lobe. The findings from this case suggested that seizures in patients with NKH result from hyperglycemia-induced BBB damage [115]. Navasardyan et al. suggested that diabetes leads to diminished BBB integrity and increased permeability, which allows the infiltration of harmful and infectious pathogens (e.g., *Mycobacterium tuberculosis*) and induces conjugate meningitis [116].

**Table 1:** Summary of studies on blood-brain barrier (BBB) impairment-related markers and brain complications in diabetic patients.

Author and year	Type of Research	Follow-up time (year)	sample size	BBB damage-related markers	Detailed description
**Cognitive impairment**					
Albert Puig-Pijoan2024 [66]	CC	3	334	Log-Qalb	With every 10% increase in log-Qalb levels, the risk of DCI progression increased by 8%
Xiaorui Tian2024 [67]	CC	1.4	295	Qalb	Patients with AD exhibited the highest levels of QAlb compared to patients with MCI and SCD. After a mean follow-up of 17 months, 117 patients (51.31%) were identified with progression of cognitive decline, and QAlb was a significant predictor of DCI progression.
Shorena Janelidze2017 [69]	CC	5.7	1015	Qalb	BBB permeability is increased in major dementia diseases. Elevated Qalb levels are associated with high concentrations of ICAM-1, VCAM-1, and VEGF in the cerebrospinal fluid.
Jinghuan Gan2023 [68]	CS	/	95	Qalb	Ratios of Aβ1-42/Aβ1-40 or t-tau/Aβ1-42 mediate the association between Qalb and GHb
Michelle M Mielke2025 [80]	CC	8-12	758	Plasma GFAP and NfL	In an elderly cohort of T2DM patients with overweight or obesity, higher plasma NfL and GFAP levels were associated with cognitive decline and an increased likelihood of cognitive impairment 8-12 years later.
J M Starr 2003 [71]	CS	/	20	Gd-DTPA enhancement	The signal intensity curves indicated a greater increase in brain signal intensity in the diabetic group compared to controls within the first 15 minutes following Gd-DTPA administration.
Ying-Chen Chen2022 [75]	CS		37	K^trans^	T2DM patients show significantly increased K^trans^, especially in the CSVD subgroup with a high dementia prevalence
Salwa S Hosny 2019 [74]	CS	/	90	sVCAM-1	Elderly T2DM patients with MCI exhibit significantly higher sVCAM-1 levels compared to T2DM patients without cognitive impairment and healthy controls.
Betul Sumbul-Sekerci2025 [121]	CS	/	183	sVCAM-1	VCAM-1 levels were significantly lower in patients with T2DM compared to the control group and the prediabetes group.
**Stroke**					
Junxia Niu2025 [122]	CS		94	K^trans^	In patients with ischemic stroke, those with diabetes exhibited significantly higher K^trans^ values compared to non-diabetic patients, along with elevated enhancement ratio measured from 3D-SPACE T1-weighted imaging.
Xinfeng Yu2016 [87]	CS	/	62	PE	Patients with both diabetes and stroke exhibited significantly increased PE.
Yonatan Serlin2016 [91]	CC	10	2982	Retinal microvascular lesions assessed by fluorescein angiography	The incidence of stroke, epilepsy, and mental disorders is higher in patients with proliferative diabetic retinopathy compared to those with non-proliferative diabetic retinopathy.
Takahiko Kawamura2006 [88]	CC	3	179	sICAM-1, sE-selectin and sVCAM-1	At baseline, patients with diabetes and asymptomatic cerebral infarction showed higher levels of sICAM-1, sE-selectin, and sVCAM-1 compared to those without infarction. Additionally, T2DM patients with baseline sICAM-1 levels >260 μg/L had an increased risk of developing stroke.
Akio Kanai2008 [89]	CC	8	179	sICAM-1	Patients with T2DM and cerebral infarction show significantly elevated sICAM-1 levels.
**Depression**					
Thanh Tan Nguyen2010 [107]	CS		82	Retinal vascular caliber measured from digital photographs	Compared with healthy controls and patients with T2DM alone, those with T2DM and comorbid major depression show more pronounced retinal arteriolar dilation.
Malgorzata Gorska-Ciebiada2015 [123]	CS	/	219	sICAM-1, sVCAM-1, and sE-selectin	In elderly diabetic patients, those with both MCI and depressive symptoms exhibit significantly higher levels of sICAM-1, sVCAM-1, and sE-selectin compared to controls.
Jean-Pierre S. Laake2014 [101]	CS		1790	VEGF	depressive symptoms measured by the continuous PHQ-9 score are positively correlated with VEGF levels.
Vera Novak2011 [124]	CS		147	sVCAM and sICAM	Patients with diabetes exhibit greater vascular reactivity, more cortical atrophy, and depression. sVCAM and sICAM are associated with increased vascular contraction, reduced vasodilation, and increased cortical atrophy in the frontal, temporal, and parietal lobes.
Christian Herder2017 [100]	CS		434	sICAM-1	sICAM-1 is positively correlated with depressive symptoms in patients with T1DM.

CC: cohort studies, CS: Cross-sectional studies, CR: case reports, Qalb: cerebrospinal fluid (CSF)/serum albumin quotient, ICAM-1: intercellular cell adhesion molecule-1, VCAM-1: vascular cell adhesion molecule-1, VEGF: Vascular endothelial growth factor, Gd: Gadolinium, MCI: Mild cognitive impairment, SCD: Subjective cognitive decline, AD: Alzheimer's disease, MRI: Magnetic Resonance Imaging, GHb: Glycosylated hemoglobin or glycated hemoglobin, PE: parenchymal enhancement, PDR: proliferative diabetic retinopathy.

Although animal experiments have shown significant BBB damage (e.g., pericyte loss, increased pathological neovascularization, microglial activation, and IgG leakage) in diabetic mice with Parkinson’s disease, there is no clinical evidence linking BBB dysfunction to Parkinson’s disease [117]. Further clinical studies are needed to explore the impact of BBB damage in patients with diabetes-related brain complications. Evidence suggests that anxiety is a diabetes-related brain complication [119]. In addition, preclinical evidence has indicated that anxiety is associated with BBB damage in female rats [120]. However, no clinical studies have confirmed the association between BBB damage and anxiety in patients with diabetes, highlighting the need for further clinical investigations.

### 3.5 Mini conclusion

The aforementioned clinical evidence indicates that the key role of BBB damage in diabetes-related brain complications has been widely recognized. BBB permeability is strongly correlated with DM-related cognitive impairment and can predict disease risk and progression [66]. In addition, BBB damage not only exacerbates early brain injury after stroke but also significantly increases the incidence of stroke and poor prognosis in patients with diabetes [87]. Finally, in patients with diabetes showing depression, BBB leakage induced by inflammation and insulin resistance is also reflected by serum biomarker levels (such as sICAM-1, S100B, and VEGF) [101, 123], and it interacts with the risk of cognitive impairment, stroke, and other diabetes-related brain disorders.

However, some controversial issues require further investigation. First, although various methods are available for BBB evaluation, including the measurement of biochemical biomarkers (such as QAlb and S100B) and imaging (Gd-DTPA enhancement, K^trans^ values, and perfusion CT findings), differences in sensitivity among these techniques may lead to discrepancies in research conclusions. Therefore, establishment of a unified multimodal assessment system and standardized evaluation criteria to improve the reproducibility of conclusions requires further consideration. Second, although some longitudinal studies have confirmed research conclusions, many cross-sectional studies have been conducted, and some of them have been unable to establish a causal relationship between BBB damage and related diseases and may have errors in their findings. BBB damage is typically an early pathological change [75] and apparent biological phenotypes of the disease may gradually emerge in its later stages [125]. Therefore, there may be a temporal difference between BBB damage and the onset of clear biological phenotypes, and further longitudinal studies with more precise designs are required to establish a clearer cause-and-effect relationship.

## 4. Pathological mechanisms of BBB damage in diabetic-related brain diseases

Hyperglycemia represents the primary insult to BBB. A number of phenotypic studies have demonstrated that hyperglycemia leads to increased BBB permeability [126-128] and impaired transport function [129-131]. Both of these effects appear to worsen over time [65]. These manifestations of BBB damage are primarily driven by a range of molecular mechanisms induced by hyperglycemia, particularly inflammation, oxidative stress, AGE accumulation, and high-mobility group box 1 (HMGB1) activation. These processes act synergistically to compromise BBB damage and contribute to the development of diabetes-related brain complications.

### 4.1 Oxidative stress

Hyperglycemic conditions can induce oxidative stress, to which the BBB is highly sensitive [132]. During hyperglycemia, glucose influx disrupts cellular energy metabolism and contributes to mitochondrial dysfunction. This disruption triggers an increase in the production of reactive oxygen species (ROS). Increased ROS further impair glucose metabolism, resulting in a vicious cycle [133, 134]. Finally, increased oxidative stress decreases the functionality of BECs [135], disrupts TJs [136], causes pericyte loss [54], and damages the actin cytoskeleton [137]. Collectively, these changes compromise BBB integrity leading to increased permeability and brain injury.

Animal studies have shown that when diabetes lasts for 3 weeks, significant oxidative stress occurs, as evidenced by reduced glutathione levels and higher levels of 3-nitrotyrosine, 4-hydroxy -2-trans-nominal, and superoxide dismutase (SOD) [138]. Meanwhile, inhibition of mitochondrial carbonic anhydrase in diabetic mice alleviates oxidative stress in the brain [138, 139], rescues pericyte loss, and reduces BBB permeability [140]. In in vitro studies, hyperglycemia promoted oxidative stress by increasing ROS levels in endothelial cells and activating SOD and catalase [135, 141, 142]. Although low hydrogen peroxide (H_2_O_2_) concentrations promote BEC proliferation, migration, and tube formation, higher concentrations (H_2_O_2_>100 μM) increase BEC permeability and reduce TJ localization [136]. The actin cytoskeleton undergoes alterations due to stress fiber contraction, resulting in gaps between endothelial cells. Oxidative stress affects the actin cytoskeleton, contributing to a significant increase in stress fiber formation and the loss of claudin-5, thus increasing BBB permeability [137]. Simultaneously, pericytes under HG have increased ROS and mitochondrial superoxide production, causing ATP depletion and decreased contractility, which leads to pericyte dysfunction and diminished coverage [54].

### 4.2 Inflammatory response

Hyperglycemia has been shown to induce both systemic and localized inflammatory responses, which play a critical role in disrupting the integrity of the BBB [143]. Animal studies have confirmed that both type 1 and type 2 diabetic mice exhibit elevated inflammatory factors in the brain tissue, contributing to memory impairment through BBB damage [59]. Another study showed that, in a diabetic insulin-resistant mouse model, BBB dysfunction and neuroinflammation in the cortex and hippocampus occurred as early as 4 weeks after high-fat, high-fructose feeding, whereas cognitive impairment only became evident after 24 weeks [20]. This suggests that BBB dysfunction related to inflammation may precede overt neurodegeneration and cognitive impairment [20]. In cellular experiments, hyperglycemic conditions prompted BECs to release inflammatory factors, e.g., TNFα, IL-6, MMP-2, and MMP-9, which in turn induced BEC damage and BBB breakdown [141, 144].

Inflammation damages BBB through several mechanisms: (a) activation of BECs involved in BBB formation and downregulation of TJs (claudin-5, occludin, ZO-1) expression [145, 146], (b) upregulated expression of inflammation-related factors and adhesion molecules (VCAM-1, ICAM-1, MMPs, and pronflammatory cytokines) [141, 145, 147], (c) an increase in reactive astrocytes [148], (d) damage to the basement membrane [149], and (e) a significant decline in P-GP efflux activity [150].

### 4.3 Advanced glycation end-products (AGEs) accumulation

Chronic hyperglycemia leads to the generation of AGEs and upregulation of their associated receptor (RAGE). This constitutes a mechanistic link between diabetes and its associated brain complications [151]. Clinical studies have shown that individuals with both T2DM and Alzheimer’s disease exhibit significantly elevated levels of AGEs in the brain [152]. Recent studies have confirmed the effects of AGEs on BBB, e.g., TJ disruption, pericyte dysfunction, MMP upregulation, and pathological changes in the basement membrane [153]. AGEs can downregulate claudin-5 expression in BECs by upregulating tumor growth factor beta-1 (TGF-β1) [154]. Furthermore, under hyperglycemic conditions and elevated AGE levels, ICAM and VCAM expressions were increased in BECs; this enhanced leukocyte adhesion and migration to BMECs monolayers. Downregulation of integrin α1, PDGF-R1β, and Cx-43 in pericytes, suggesting cellular dysfunction, has also been observed [53]. The accumulation of AGEs also increases fibronectin levels in pericytes [155] and upregulates MMP-2 [155] and MMP-9 [156] activities in rats, driving pathological changes in the basement membrane. The AGE-RAGE axis is one of the pathogenic pathways in diabetic brain injury. Through interaction with RAGE, AGEs can induce BBB damage by activating neuroinflammation [157].

### 4.4 HMGB1 signaling axis activation

The activation of the HMGB1 signaling axis under hyperglycemic conditions is another important contributor to BBB damage. HMGB1, a nuclear DNA-binding protein, is elevated in patients and mice with diabetes [158, 159]. Overactivation of HMGB1 can mediate downstream signaling pathways and interact with RAGE, TLR4, NLRP3, NMDAR, and other molecules, affecting the BBB by promoting neuroinflammation [160], disrupting TJs [161], inducing oxidative stress [162], and leading to endothelial barrier injury [163]. These responses eventually increase BBB permeability, causing various diabetes-related brain complications such as stroke and cognitive impairment.

Thus, HMGB1 is a potential therapeutic target for diabetes-related brain complications. Suppression of HMGB1-related signaling can inhibit amyloid beta (Aβ) accumulation in the hippocampus and improve BBB permeability in aging mice, leading to the restoration of cognitive functions [164]. Additionally, HMGB1 inhibition modulates microglia-mediated neuro-inflammation and improves BBB integrity, thereby preventing cerebral ischemic injury [165]. One study found a significant positive correlation between elevated clinical serum HMGB1 levels and depression severity [166]. In a depressed C57BL/6 mouse model, depression-associated spontaneous behavior was alleviated by inhibiting the colonic HMGB1-RAGE-TLR4 signaling axis [167]. Nevertheless, till date, there has been no direct evidence linking HMGB1 to depression via BBB damage.

### 4.5 Mini conclusion

Based on the discussed pathological mechanisms, hyperglycemia is the primary driving factor for BBB damage in diabetes-related brain complications. Oxidative stress, inflammation, AGEs accumulation, and HMGB1 signaling activation synergistically contribute to BBB damage. These processes promote TJ protein degradation, dysfunction of transport proteins, alterations in the basement membrane structure, activation of reactive astrocytes, loss of pericytes, and dysfunction of endothelial cells. Ultimately, these pathological responses lead to significantly increased BBB permeability, BBB dysfunction, and various brain complications, including cognitive impairment, stroke, and depression. In these studies, the pathological mechanisms did not occur in isolation but amplified each other through multiple positive feedback loops, collectively exacerbating BBB damage. This highlights the need for multitarget combination therapies based on BBB damage.

**Figure 2.**
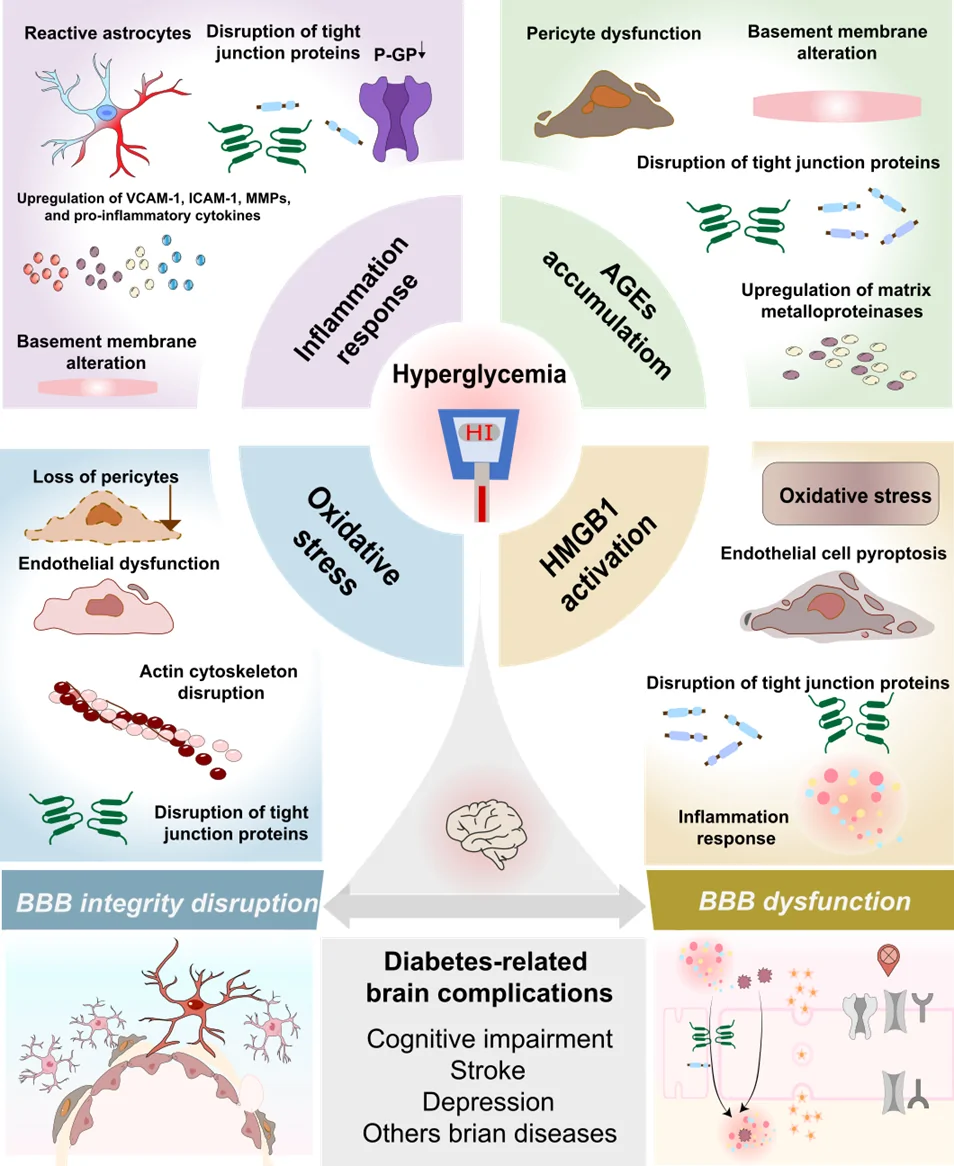
**Pathological mechanisms underlying blood-brain barrier (BBB) disruption in diabetes-related brain complications**. Hyperglycemia and pathological factors such as inflammation, oxidative stress, accumulation of advanced glycation end products (AGEs), and activation of the high-mobility group box 1 (HMGB1) signaling axis collectively contribute to structural damage and functional disruption of BBB, ultimately leading to diabetes-associated brain complications. P-GP: P-glycoproteins, AGEs: advanced glycosylation end products, HMGB1: the high mobility group protein 1, VCAM-1: vascular cell adhesion molecule-1, ICAM-1: intercellular adhesion molecule-1, MMPs: matrix metalloproteinases, BBB: blood-brain barrier

## 5. Interventions to repair BBB damage in diabetic-related brain complications

BBB damage is a common phenotypic feature and a potential therapeutic target in diabetes-related brain complications. Lifestyle improvements and medications can be effective and exert a combined therapeutic effect.

### 5.1 Lifestyle interventions

The impairment of BBB integrity and permeability can be partially reversed by physical activity and a healthy diet. A study showed that aerobic exercise can improve the oxidant-antioxidant imbalance in subjects with obesity and reduce the levels of serum S100β and neuron-specific enolase (NSE), findings suggesting an improvement in BBB [168]. Animal experiments showed that claudin-5 expression was upregulated in the striatum of diabetic rats after exercise, while synaptic plasticity was restored in the prefrontal cortex, leading to improved short-term memory [169, 170]. Similarly, running exercises alleviated depressive symptoms in mice by activating the AMPK-NF-κB/STAT3 signaling pathway, which can maintain the balance between M1 and M2 microglia and alleviate neuroinflammation [171]. Lu et al. found that swimming protected BBB integrity, reduced neuroinflammation, and preserved cognitive function in diabetic rats by downregulating fibrinogen levels and astrocyte activation [172]. Research has also found a positive effect of exercise on BBB integrity and brain edema in post-stroke rats [173].

Dietary patterns strongly correlate with DM. Several clinical studies have found that an inflammatory diet is independently associated with an increased risk of T2DM and may also elevate the mortality risk in patients with diabetes [174-176]. A Mediterranean diet can improve fasting blood glucose levels, HbA1c levels, and insulin sensitivity in patients with T2DM [177]. Similarly, a low-carbohydrate diet can promote weight loss; reduce blood glucose, IL-1Ra, and IL-6 levels; and improve the inflammatory status in patients with T2DM [178]. After 12 weeks of intermittent fasting, mice fed a high-fat diet showed memory recovery, coinciding with increased claudin-5 and ZO-1 expression and reduced extravascular albumin leakage; these findings suggested that intermittent fasting reduces BBB leakage [179]. Similarly, meta-analyses have highlighted that physical activity and dietary changes are beneficial for alleviating cognitive dysfunction [180-183], stroke [184, 185], and depression [186, 187] in patients with DM.

### 5.2 Clinical medications

#### 5.2.1 Hypoglycemic agents

Many studies have reported the beneficial effects of hypoglycemic agents on diabetes-related brain complications; these were also found to improve BBB permeability and transport function. However, the results of some of these studies remain controversial.

Meta-analyses have suggested that metformin benefits patients with DM who show cognitive impairment [188] and stroke [189], but not those who show depression [190]. However, in a recent cohort study of 89,711 individuals with DM, metformin was found to reduce the risk of anxiety and depression [191]. Metformin reduces neuroinflammation and gliosis while enhancing BBB permeability and transport function, mainly by (a) protecting endothelial cells from emergency stimuli and infiltration of inflammatory factors [192-195], (b) increasing expression of ZO-1 and occludin to maintain BBB integrity [196], (c) inhibiting cortical microglia activation and decreasing proinflammatory factors [197], (d) improving pericyte coverage [198], and (e) suppressing the RAGE signaling pathway and upregulating LRP1 [199].

Dipeptidyl peptidase 4 (DPP-4) inhibitors and glucagon-like peptide-1 (GLP-1) receptor agonists (GLP-1RA) also protect CNS by increasing the active form of GLP-1 [200, 201]. GLP-1RA is expressed in cognitively relevant CNS regions, e.g. the hippocampus, prefrontal cortex, and hypothalamus. Thus, elevated GLP-1RA levels play a vital role in restoration of BBB integrity and maintenance of permeability [202-204]. The DPP-4 inhibitor rosiglitazone reduces the pathological vasculature and increases pericyte density, thus restoring BBB integrity and reversing T2DM-induced BBB leakage [205]. Meta-analyses reported positive effects of GLP1-RA on cognitive impairment [206], stroke [207, 208], and depression [209]. As for DPP-4 inhibitors, although it may benefit cognitive function [210], their use in cases of stroke [211, 212] and depression [213, 214] remains controversial.

Meta-analyses have indicated that sodium-glucose transporter-2 (SGLT2) inhibitors (SGLT2is) diminished the risk of brain diseases such as dementia [206], stroke [215], and depression [214] in patients with diabetes and may be more efficient than other hypoglycemic agents. SGLT2 is present in the brain parenchyma and BBB [216], where it interacts with SGLT-2i, thereby offering neuroprotective effects [217, 218]. Animal experiments demonstrated that canagliflozin downregulates VEGF-A and upregulates ZO-1 in diabetic mice, significantly reducing pathological cerebral neovascularization and restoring BBB permeability [219].

Thiazolidinediones enhance BBB permeability and transport functions. An in vitro study confirmed that pioglitazone reduces BBB permeability of sodium fluorescein and ICAM-1 expression [220]. Furthermore, pioglitazone and rosiglitazone were found to upregulate LRP1 expression and recover Aβ uptake [221, 222]. With regard to clinical settings, meta-analyses have suggested that thiazolidinediones reduce the risk of dementia [223], depression [190], and stroke [224] among patients with DM.

However, the efficacy of sulphonylureas in diabetes-related brain complications remains controversial. Some meta-analyses have revealed that sulfonylureas increase the risk of dementia in patients with diabetes [206], and that the risk is significantly higher than that with other hypoglycemic agents [225]. However, another study found no significant effect of sulfonylureas on the increased risk of stroke [226]. A retrospective study suggested that sulfonylureas may be neuroprotective and alleviate adverse neurological outcomes in patients with diabetes showing aneurysmal subarachnoid hemorrhage [227]. Variations in study criteria and literature volume may explain these discrepancies. Additionally, induced hypoglycemia may be a key reason for its contribution to CNS diseases [206]. Animal experiments revealed that glimepiride protects BBB by restoring pericyte coverage and reducing microglial activation [205]. Glibenclamide was also found to prevent cognitive impairment and depression in mice [228]. Possible mechanisms include inhibition of NLRP3 inflammasome activation in BMECs, boosting of ZO-1 expression, and a decrease in inflammatory cytokine levels, resulting in preserved BBB integrity and permeability [229].

Insulin treatment improves the diabetes-impaired BBB in a dose-dependent manner, both in terms of barrier and transporter functions. Insulin treatment reduces the monolayer permeability of hCMEC/D3 cells to IgG, increases brain transepithelial electrical resistance, and elevates the levels of the transporter protein ABCB1 [230]. In diabetic rats, insulin may increase occludin, claudin-5, and ZO-1 levels in BBB while enhancing P-GP levels through the ROS/AGE/RAGE/NF-κB signaling pathway, leading to reduced BBB permeability and restored transport function [231, 232]. Different routes of administration have varying clinical effects. A recent meta-analysis showed that insulin injections have no significant effect on the risk of dementia in patients with diabetes [223], likely because of hypoglycemic risk factors [206]. Another meta-analysis proposed the use of intranasal insulin (INI) to improve cognitive function, especially in patients with Alzheimer's disease (AD) or mild cognitive impairment (MCI) [233]. Compared with traditional multiple daily insulin injections, insulin pump usage reportedly decreased the incidence and mortality rate of severe cardiovascular diseases, such as coronary heart disease and stroke, in patients with diabetes [234]. A meta-analysis also showed that although insulin injections improve diabetes-related distress, they do not affect depression and anxiety [235].

#### 5.2.2 Antihypertensive agents

Clinical studies have shown that intensive blood pressure control can reduce the risk of cognitive impairment [236] and stroke [237] in patients with DM.

**Table 2:** Effect of different interventions on blood-brain barrier damage and comorbidities in diabetes mellitus.

Interventions	BBB-related indicators	Diabetic comorbidities		
		Cognitive impairment	Stroke	Depression
**Lifestyle factors**				
Exercise	Claudin-5⬆ [169]	A meta-analysis including 12 trials found a beneficial effect [180].	A meta-analysis including 43 trials found a beneficial effect [184].	A meta-analysis including 17 trials found a beneficial effect [186].
Diet	Claudin-5 and ZO-1⬆, extravasated albumin ⬇ [179]	Meta-analyses have shown beneficial effects of dietary interventions in both diabetes [181] and cognitive impairment [182, 183], but no studies related to comorbidities.	A meta-analysis including 41 trials found a beneficial effect [185].	A meta-analysis including 13 trials found a beneficial effect [187].
**Hypoglycemic agents**				
Metformin	LRP1⬆[199]	A meta-analysis including 28 trials found a beneficial effect [247].	A meta-analysis including 21 trials found a beneficial effect, but not in combination with other hypoglycemic agents [189].	A meta-analysis that included five trials found no significant effect [190]. However, the latest cohort study, which included 89,711 patients, found a beneficial effect of metformin [191].
GLP-1RA	Occludin and AQP4⬆,EB, MMP-2 and MMP-9⬇[203, 204]	A meta-analysis including 5 trials found no beneficial effect[248]. However, a beneficial effect was found in the latest network meta-analysis including 27 trials [206].	Two meta-analyses both found beneficial effects [207, 208].	A meta-analysis including 5 trials found a beneficial effect [209].
DPP-4 inhibitors	IgG and albumin⬇[205]	A meta-analysis including 10 trials found a beneficial effect [210].	Meta-analysis suggested no significant effect [211, 212].	A case-control study suggested a beneficial effect [213], but another cohort study suggested it was not significant [214].
SGLT-2i	VEGF-A and EB⬇, ZO-1⬆[219]	A network meta-analysis including 27 trials found a beneficial effect [206].	A meta-analysis including 12 trials found a beneficial effect [215].	A cohort study suggested a beneficial effect [214].
Thiazolidinedione drugs	LRP1⬆, Fluorescein sodium⬇, Increased Aβ efflux from the BBB and decreased Aβ influx [220-222]	A network meta-analysis including 27 trials found a beneficial effect [206].	A meta-analysis including 3 trials found a beneficial effect [224].	A meta-analysis including 19 trials found a beneficial effect [190].
Sulfonylureas	Pericyte coverage⬆, microglia activation, IgG, and albumin ⬇[205]	A network meta-analysis including 27 studies found an increased risk of dementia [206].	A meta-analysis including 108 trials suggested a significantly higher risk than other hypoglycaemic agents [225]. Another meta-analysis that included 47 trials suggested no significant effect [226].	A randomized trial suggested no significant effect [249].
Insulin	Occludin⬆, claudin-5⬆, ZO-1⬆, P-GP⬆, Rho123⬇,IgG⬇, and TEER⬆[230-232]	A meta-analysis of 27 trials suggested no significant effect [223], and a meta-analysis including 29 trials suggested a beneficial effect of intranasal insulin [233].	An observational study including 18,168 patients with type 1 diabetes suggested a beneficial effect of insulin pumps [234].	A meta-analysis including 9 trials suggested a reduction in diabetes-related pain, but no significant effect on depression [235].
**Antihypertensive agents**				
Telmisartan	ZO-1, occludin, claudin-3, claudin-5, and VE-Cadherin⬆,MMP-2 and MMP-9⬇[238]	A cohort study of 65,511 patients suggested a beneficial effect [250].	A retrospective study of 54,186 patients suggested a beneficial effect [251].	No relevant clinical studies.
Candesartan	EB⬇, TEER⬆[239, 240]	No relevant clinical studies.	No relevant clinical studies.	No relevant clinical studies.
Nifedipine	EB⬇[241]	No relevant clinical studies.	No relevant clinical studies.	No relevant clinical studies.
**Lipid-lowering agents**				
Probucol	IgG⬇,occludin-1⬆[15]	No relevant clinical studies.	No relevant clinical studies	No relevant clinical studies.
Statin	250-, 70-, and 40-kDa dextrans volume of distribution⬇, Annexin A1, claudin-3, and VE-cadherin⬆[242, 243]	A meta-analysis including 15 trials showed a beneficial effect [244].	A meta-analysis including 9 trials showed a beneficial effect [245].	A meta-analysis including 90 trials suggested a non-significant effect [246].

ZO-1: zonula occludens-1, LRP1: lipoprotein receptor-related protein 1, VEGF-A: vascular endothelial growth factor-A, MMP-2: matrix metalloproteinase-2, MMP-9: matrix metalloproteinase-9, EB: Evans blue extravasation, TEER: transepithelial electrical resistance, P-GP: P-glycoproteins, Rho123: rhodamine 123, IgG: immunoglobulin G

Some studies have supported the potential benefits of renin-angiotensin-aldosterone system inhibitors and calcium channel blockers in the treatment of diabetes-associated depression; however, there are no relevant clinical studies till date. The efficacy of antihypertensive agents against BBB damage in diabetes has also been investigated in animal studies. Telmisartan can alleviate BBB damage to improve diabetes-induced cognitive impairment via the activation of peroxisome proliferator-activated receptor γ [238]. A study showed that candesartan significantly reduced BBB permeability and lowered lipid peroxide levels [239], consistent with the findings of another study [240]. A study also showed that nifedipine significantly reduced Evans blue extravasation, indicating improved BBB permeability [241].

#### 5.2.3 Lipid-lowering agents

Probucol tablets exhibit both lipid-lowering and anti-lipid peroxidation effects. Research indicated that probucol tablets not only improved insulin resistance and peripheral inflammation in diabetic mice but also prevented IgG efflux in BBB and upregulated occludin-1 expression in BECs, driving a reduction in cognitive impairment [15]. Research on statins has highlighted that rosuvastatin and simvastatin decrease the distribution volumes of dextrans in diabetic rats, suggesting restoration of BBB permeability [242]. Another study of diabetic rats confirmed that rosuvastatin upregulates biomarkers of BBB integrity such as annexin A1, claudin-3, and VE-cadherin, restoring cognitive function [243]. Meta-analyses of clinical studies have indicated that statins may slow cognitive decline [244] and lower the stroke risk [245], while showing no association with increased depression risk [246], among patients with DM.

### 5.3 Preclinical therapeutic approaches

#### 5.3.1 Targeted nanomedicines in diabetes-related brain complications

Targeted nanomedicines represent a treatment modality that utilizes nanoparticles as drug carriers for delivery. Research has increasingly explored these therapeutics in conditions associated with BBB damage, with the aim of mitigating BBB inflammatory responses and enhancing drug delivery across BBB to improve pharmacokinetics and intracerebral drug concentrations. Recent research indicated that in mice with ischemic stroke, lipid nanocarriers that target VCAM significantly enhanced drug localization within the compromised BBB region, leading to a 62% reduction in the volume of cerebral infarcts compared to that with non-targeted controls [252]. Anti-VCAM/liposomes also specifically target the inflamed brain, binding to endothelial cells, reducing BBB permeability, and attenuating TNF-α-induced brain edema [253]. Other studies have focused on creating nanoparticle delivery systems to transport medications across BBB. This strategy aims to increase the intracerebral concentrations of therapeutic agents and enhance their efficacy. For instance, a nanoparticle platform using small interfering RNA (siRNA) can triple drug concentrations in mice with traumatic brain injuries [247]. Another example is a nanoparticle-targeting ginsenoside, Rg1, capable of penetrating the BBB effectively and reducing the cerebral infarction volume [254]. Quercetin-conjugated superparamagnetic iron oxide nanoparticles show significant efficacy against diabetes-related cognitive impairment; however, their specific mechanisms remain unexplored [255].

#### 5.3.2 Other agents

Some potential treatments, e.g., natural compounds and antioxidants, focus on the effects of BBB damage in DM. Allen et al. found that hyperglycemia may impair BBB integrity through oxidative stress, and different antioxidants such as NAD(P)H oxidase inhibitors, vitamin C, free radical scavengers, antioxidant enzymes, and different SOD mimetics can alleviate oxidative stress and lower hyperglycemia to restore BBB integrity [135]. Another study also found that antioxidant polyphenols can reduce high glucose-mediated cerebral infarct volume and hemorrhagic transformation, mainly by weakening redox reactions, lowering ROS levels, and inhibiting NOX activation to protect cerebral endothelial cells [256]. Sesamol, a natural antioxidant, upregulates TJ expression in BBB and reduces barrier permeability in diabetic rats [257]. T The major lignan (SDG) in flaxseed is a natural compound with anti-inflammatory properties. Studies have confirmed that SDG reduces leukocyte adhesion and migration across BBB, downregulates VCAM-1 expression, and restores BBB permeability [258]. Lauric acid, a naturally occurring fatty acid, has also been shown to reduce infarct volume and brain edema in post-stroke diabetic mice by reducing BEC death and stabilizing BBB barrier functions [259]. Likewise, several inhibitors have shown protective effects on BBB in patients with diabetes; these include the RAGE-specific inhibitor FPS-ZM1 [260], the NLRP3 inhibitor MCC950 [261], the HDAC3 inhibitor RGFP966 [262], and the NF-κB inhibitor pyrrolidine dithiocarbamate [263].

Although many studies have demonstrated a relationship between the gut-brain axis, diabetes, and its associated brain complications [264], conclusions regarding the effects of prebiotics and probiotics remain inconsistent. One study found that fructo-oligosaccharide (FOS) treatment increased the abundance of *Bifidobacterium* in the gut of db/db mice and improved neuroinflammation and BBB integrity; however, it failed to alleviate anxiety-like behavior and spatial memory deficits [265]. In contrast, Morshedi et al. reported that supplementation with inulin, *Lactobacillus plantarum*, or a combination of both improved depressive and anxiety-like behaviors in diabetic rats [266]. Similarly, Hosseinifard et al. found that supplementation with *L. plantarum* and inulin modulated the composition of the gut microbiota, leading to reduced GFAP and glial cell-line derived neurotrophic factor (GDNF) levels in the amygdala [267]. This reduction was associated with an improvement in anxiety-like behavior in diabetic rats. However, no significant effects were observed in the prefrontal cortex. Further research is needed to determine whether prebiotics and probiotics influence diabetes-related brain complications through BBB regulation.

### 5.4 Mini conclusion

BBB damage is a potential therapeutic target in diabetes-related brain complications such as cognitive impairment, stroke, and depression. Lifestyle interventions, various clinical medications, and emerging nanocarrier drug delivery systems can repair diabetes-induced BBB damage by varying degrees, thereby lowering the risk of such complications. However, some aspects deserve further discussion. First, although some studies have evaluated the role of hypoglycemic agents in improving BBB integrity and reducing diabetes-related brain complications, the results remain controversial, particularly for DPP-4 inhibitors [210-214], sulfonylureas[206, 225, 226, 249], and insulin[223, 233-235]. This suggests the need to focus on differences between basic research and clinical practice as well as the impact of potential adverse side effects on the human body. We also found differences in the effects of various insulin formulations (e.g., INI improves cognitive function in patients with diabetes) [223, 233]; therefore, methods to optimize the use of different forms deserve further study. Second, preclinical studies highlight the advantages of rigorous blood pressure and lipid control for managing diabetes-related brain complications [238, 243]; however, clinical evidence remains limited and requires further examination. Third, nanocarrier-based drug delivery systems are being increasingly studied as emerging strategies for improving BBB integrity [252, 253]. However, further research is needed to confirm the safety of these treatments and elucidate their specific mechanisms, which will help promote their clinical translation.

## 6. Final conclusion and prospects

Diabetes-related brain complications such as cognitive impairment, stroke, and depression often occur simultaneously or sequentially. Their complex pathological mechanisms and the use of multiple medications pose significant challenges in clinical management. Although some studies have focused on changes in the structure and function of BBB in cases of diabetes with cognitive impairment [268] and stroke [17], elated clinical evidence and interventions remain unclear. This paper provides a review of the structure and function of BBB and analyzes the mechanisms by which pathological factors such as hyperglycemia, oxidative stress, inflammation, AGEs, and HMGB1 contribute to BBB damage. It also summarizes the evidence generated by clinical and animal studies on lifestyle and pharmacological interventions for repairing BBB and reducing brain complications. We found that BBB permeability markers such as Gd-DTPA enhancement and QAlb and S100β levels are significantly associated with cognitive decline, stroke outcomes, and depressive symptoms. However, current assessment methods are not standardized, and most intervention studies are based on small sample sizes or short-term durations. Larger-scale, more rigorously designed clinical trials are urgently required for further validation.

Future research should focus on the following directions: integration of heterogeneous data such as magnetic resonance imaging and other imaging data with biological markers through machine learning models can improve diagnostic sensitivity and specificity. Additionally, focusing on subclinical features before disease onset to predict high-risk patients before the appearance of symptoms enables early intervention. Implementation of personalized screening for high-risk diabetic populations and testing of preventive interventions (e.g., INI and microbiome modulation) can help maintain BBB integrity before significant neurodegeneration occurs. For patients with BBB damage and diabetes-related brain complications, treatment precision needs to be enhanced by selection of glucose-lowering and antihypertensive medications that are more favorable for BBB repair, in conjunction with lifestyle interventions. Finally, preclinical drugs targeting BBB, such as nanomedicines and inhibitors, require further research for evaluation of their safety and ability to cross BBB.

## Data Availability

No datasets were generated or analyzed during the current study.

## Copyright of Figures

Copyright © 2025 [Caiyi Long]. All figures in this manuscript are licensed under the GNU General Public License. This program is free software, which can be redistributed and/or modified under the terms of the GNU General Public License as published by the Free Software Foundation, either version 3 of the License, or any later version. See the GNU General Public License for more details: www.gnu.org/licenses/.

## References

[1] (2021). Diabetes is "a pandemic of unprecedented magnitude" now affecting one in 10 adults worldwide. Diabetes Res Clin Pract, 181:109133. 10.1016/j.diabres.2021.109133 34801177

[2] van Sloten TT, Sedaghat S, Carnethon MR, Launer LJ, Stehouwer CDA (2020). Cerebral microvascular complications of type 2 diabetes: stroke, cognitive dysfunction, and depression. Lancet Diabetes Endocrinol, 8:325-336. 10.1016/S2213-8587(19)30405-X PMC11044807 32135131

[3] McCall AL (1992). The Impact of Diabetes on the CNS. Diabetes, 41:557-570. 10.2337/diab.41.5.557 1568525

[4] Patel SS, Udayabanu M (2017). Effect of natural products on diabetes associated neurological disorders. Rev Neurosci, 28:271-293. 10.1515/revneuro-2016-0038 28030360

[5] Xue M, Xu W, Ou YN, Cao XP, Tan MS, Tan L, et al. (2019). Diabetes mellitus and risks of cognitive impairment and dementia: A systematic review and meta-analysis of 144 prospective studies. Ageing Res Rev, 55:100944. 10.1016/j.arr.2019.100944 31430566

[6] Jansen van Vuuren JM, Pillay S (2018). Major depressive disorder in patients with diabetes mellitus in Pietermaritzburg, South Africa. S Afr Med J, 109:58-61. 10.7196/SAMJ.2018.v109i1.13356 30606306

[7] Lau LH, Lew J, Borschmann K, Thijs V, Ekinci EI (2019). Prevalence of diabetes and its effects on stroke outcomes: A meta-analysis and literature review. J Diabetes Investig, 10:780-792. 10.1111/jdi.12932 PMC6497593 30220102

[8] Sarwar N, Gao P, Seshasai SR, Gobin R, Kaptoge S, Di Angelantonio E, et al. (2010). Diabetes mellitus, fasting blood glucose concentration, and risk of vascular disease: a collaborative meta-analysis of 102 prospective studies. Lancet, 375:2215-2222. 10.1016/S0140-6736(10)60484-9 PMC2904878 20609967

[9] Hou H, Sun G, Duan Z, Tao L, Zhang S, Fang Q (2022). Clinical characteristics of cognitive impairment and its related risk factors in post-stroke epilepsy. Epileptic Disord, 24:677-686. 10.1684/epd.2022.1442 35792846

[10] Behl T, Kaur I, Sehgal A, Khandige PS, Imran M, Gulati M, et al. (2024). The Link Between Alzheimer's Disease and Stroke: A Detrimental Synergism. Ageing Res Rev:102388. 10.1016/j.arr.2024.102388 38914265

[11] Wal P, Kumar P, Bhardwaj H, Sharma K, Tripathi AK, Gupta A, et al. (2024). Comorbidity of Depression and Diabetes: A Literature Review on Systemic Flaws in Healthcare and the Benefits of Collaborative Diagnosis and Treatment in Primary Care Settings. Curr Diabetes Rev. 10.2174/0115733998288090240509105717 38798204

[12] Cai J, Zhang S, Wu R, Huang J (2024). Association between depression and diabetes mellitus and the impact of their comorbidity on mortality: Evidence from a nationally representative study. J Affect Disord, 354:11-18. 10.1016/j.jad.2024.03.003 38447915

[13] Echouffo-Tcheugui JB, Xu H, Matsouaka RA, Xian Y, Schwamm LH, Smith EE, et al. (2018). Diabetes and long-term outcomes of ischaemic stroke: findings from Get With The Guidelines-Stroke. Eur Heart J, 39:2376-2386. 10.1093/eurheartj/ehy036 PMC6031049 29438515

[14] Banks WA (2019). The blood-brain barrier as an endocrine tissue. Nat Rev Endocrinol, 15:444-455. 10.1038/s41574-019-0213-7 31127254

[15] Mamo JC, Lam V, Brook E, Mooranian A, Al-Salami H, Fimognari N, et al. (2019). Probucol prevents blood-brain barrier dysfunction and cognitive decline in mice maintained on pro-diabetic diet. Diab Vasc Dis Res, 16:87-97. 10.1177/1479164118795274 30156119

[16] Yamamoto M, Guo DH, Hernandez CM, Stranahan AM (2019). Endothelial Adora2a Activation Promotes Blood-Brain Barrier Breakdown and Cognitive Impairment in Mice with Diet-Induced Insulin Resistance. J Neurosci, 39:4179-4192. 10.1523/JNEUROSCI.2506-18.2019 PMC6529868 30886019

[17] Wicha P, Das S, Mahakkanukrauh P (2021). Blood-brain barrier dysfunction in ischemic stroke and diabetes: the underlying link, mechanisms and future possible therapeutic targets. Anat Cell Biol, 54:165-177. 10.5115/acb.20.290 PMC8225477 33658432

[18] Mázala-de-Oliveira T, Silva BT, Campello-Costa P, Carvalho VF (2023). The Role of the Adrenal-Gut-Brain Axis on Comorbid Depressive Disorder Development in Diabetes. Biomolecules, 13. 10.3390/biom13101504 PMC10604999 37892186

[19] Du Q, Wang YH, Zhao HQ, Yang H, Meng P, Xu YL (2016). [Damages and its mechanism of the blood brain barrier in rats with diabetes mellitus with depression]. Zhongguo Ying Yong Sheng Li Xue Za Zhi, 32:558-562. 10.13459/j.cnki.cjap.2016.06.016 29926627

[20] Takechi R, Lam V, Brook E, Giles C, Fimognari N, Mooranian A, et al. (2017). Blood-Brain Barrier Dysfunction Precedes Cognitive Decline and Neurodegeneration in Diabetic Insulin Resistant Mouse Model: An Implication for Causal Link. Front Aging Neurosci, 9:399. 10.3389/fnagi.2017.00399 PMC5717019 29249964

[21] Daneman R, Zhou L, Kebede AA, Barres BA (2010). Pericytes are required for blood-brain barrier integrity during embryogenesis. Nature, 468:562-566. 10.1038/nature09513 PMC3241506 20944625

[22] Brightman MW, Reese TS (1969). Junctions between intimately apposed cell membranes in the vertebrate brain. J Cell Biol, 40:648-677. 10.1083/jcb.40.3.648 PMC2107650 5765759

[23] Chen A, Volpato G, Pong A, Schofield E, Huang J, Qiu Z, et al. (2024). The Blood-Brain Barrier in Both Humans and Rats: A Perspective From 3D Imaging. Int J Biomed Imaging, 2024:4482931. 10.1155/2024/4482931 PMC11368551 39224835

[24] Yang Z, Lin P, Chen B, Zhang X, Xiao W, Wu S, et al. (2021). Autophagy alleviates hypoxia-induced blood-brain barrier injury via regulation of CLDN5 (claudin 5). Autophagy, 17:3048-3067. 10.1080/15548627.2020.1851897 PMC8526012 33280500

[25] Li Y, Wei JY, Liu H, Wang KJ, Jin SN, Su ZK, et al. (2022). An oxygen-adaptive interaction between SNHG12 and occludin maintains blood-brain barrier integrity. Cell Rep, 39:110656. 10.1016/j.celrep.2022.110656 35417709

[26] Fanning AS, Jameson BJ, Jesaitis LA, Anderson JM (1998). The tight junction protein ZO-1 establishes a link between the transmembrane protein occludin and the actin cytoskeleton. J Biol Chem, 273:29745-29753. 10.1074/jbc.273.45.29745 9792688

[27] Peng BH, Lee JC, Campbell GA (2003). In vitro protein complex formation with cytoskeleton-anchoring domain of occludin identified by limited proteolysis. J Biol Chem, 278:49644-49651. 10.1074/jbc.M302782200 14512431

[28] Ohtsuki S, Sato S, Yamaguchi H, Kamoi M, Asashima T, Terasaki T (2007). Exogenous expression of claudin-5 induces barrier properties in cultured rat brain capillary endothelial cells. J Cell Physiol, 210:81-86. 10.1002/jcp.20823 16998798

[29] Nitta T, Hata M, Gotoh S, Seo Y, Sasaki H, Hashimoto N, et al. (2003). Size-selective loosening of the blood-brain barrier in claudin-5-deficient mice. J Cell Biol, 161:653-660. 10.1083/jcb.200302070 PMC2172943 12743111

[30] Adams CL, Chen YT, Smith SJ, Nelson WJ (1998). Mechanisms of epithelial cell-cell adhesion and cell compaction revealed by high-resolution tracking of E-cadherin-green fluorescent protein. J Cell Biol, 142:1105-1119. 10.1083/jcb.142.4.1105 PMC2132880 9722621

[31] Itoh M, Nagafuchi A, Moroi S, Tsukita S (1997). Involvement of ZO-1 in cadherin-based cell adhesion through its direct binding to alpha catenin and actin filaments. J Cell Biol, 138:181-192. 10.1083/jcb.138.1.181 PMC2139940 9214391

[32] Armulik A, Genové G, Mäe M, Nisancioglu MH, Wallgard E, Niaudet C, et al. (2010). Pericytes regulate the blood-brain barrier. Nature, 468:557-561. 10.1038/nature09522 20944627

[33] Janzer RC, Raff MC (1987). Astrocytes induce blood-brain barrier properties in endothelial cells. Nature, 325:253-257. 10.1038/325253a0 3543687

[34] Guérit S, Fidan E, Macas J, Czupalla CJ, Figueiredo R, Vijikumar A, et al. (2021). Astrocyte-derived Wnt growth factors are required for endothelial blood-brain barrier maintenance. Prog Neurobiol, 199:101937. 10.1016/j.pneurobio.2020.101937 33383106

[35] Argaw AT, Asp L, Zhang J, Navrazhina K, Pham T, Mariani JN, et al. (2012). Astrocyte-derived VEGF-A drives blood-brain barrier disruption in CNS inflammatory disease. J Clin Invest, 122:2454-2468. 10.1172/JCI60842 PMC3386814 22653056

[36] Moore ED, Kooshki M, Metheny-Barlow LJ, Gallagher PE, Robbins ME (2013). Angiotensin-(1-7) prevents radiation-induced inflammation in rat primary astrocytes through regulation of MAP kinase signaling. Free Radic Biol Med, 65:1060-1068. 10.1016/j.freeradbiomed.2013.08.183 PMC3879043 24012919

[37] Weng Y, Chen N, Zhang R, He J, Ding X, Cheng G, et al. (2024). An integral blood-brain barrier in adulthood relies on microglia-derived PDGFB. Brain Behav Immun, 115:705-717. 10.1016/j.bbi.2023.11.023 37992789

[38] Yenari MA, Xu L, Tang XN, Qiao Y, Giffard RG (2006). Microglia potentiate damage to blood-brain barrier constituents: improvement by minocycline in vivo and in vitro. Stroke, 37:1087-1093. 10.1161/01.STR.0000206281.77178.ac 16497985

[39] Armstrong BK, Robinson PJ, Rapoport SI (1987). Size-dependent blood-brain barrier opening demonstrated with [14C]sucrose and a 200,000-Da [3H]dextran. Exp Neurol, 97:686-696. 10.1016/0014-4886(87)90125-7 2442027

[40] Li G, Fu BM (2011). An electrodiffusion model for the blood-brain barrier permeability to charged molecules. J Biomech Eng, 133:021002. 10.1115/1.4003309 21280874

[41] Lomize AL, Pogozheva ID (2019). Physics-Based Method for Modeling Passive Membrane Permeability and Translocation Pathways of Bioactive Molecules. J Chem Inf Model, 59:3198-3213. 10.1021/acs.jcim.9b00224 PMC6756154 31259555

[42] Terasaki T, Hosoya K (1999). The blood-brain barrier efflux transporters as a detoxifying system for the brain. Adv Drug Deliv Rev, 36:195-209. 10.1016/s0169-409x(98)00088-x 10837716

[43] Tournier N, Langer O (2025). Imaging the Activity of Efflux Transporters at the Blood-Brain Barrier in Neurologic Diseases: Radiotracer Selection Criteria. [J] Nucl Med. 10.2967/jnumed.124.269322 PMC12051769 40015923

[44] Kuo YC, Lu CH (2011). Regulation of endocytosis into human brain-microvascular endothelial cells by inhibition of efflux proteins. Colloids Surf B Biointerfaces, 87:139-145. 10.1016/j.colsurfb.2011.05.014 21636258

[45] Gynther M, Ropponen J, Laine K, Leppänen J, Haapakoski P, Peura L, et al. (2009). Glucose promoiety enables glucose transporter mediated brain uptake of ketoprofen and indomethacin prodrugs in rats. J Med Chem, 52:3348-3353. 10.1021/jm8015409 19402664

[46] Reichel A, Begley DJ, Abbott NJ (2000). Carrier-mediated delivery of metabotrophic glutamate receptor ligands to the central nervous system: structural tolerance and potential of the L-system amino acid transporter at the blood-brain barrier. J Cereb Blood Flow Metab, 20:168-174. 10.1097/00004647-200001000-00021 10616805

[47] Jefferies WA, Brandon MR, Hunt SV, Williams AF, Gatter KC, Mason DY (1984). Transferrin receptor on endothelium of brain capillaries. Nature, 312:162-163. 10.1038/312162a0 6095085

[48] Petralla S, Panayotova M, Franchina E, Fricker G, Puris E (2024). Low-Density Lipoprotein Receptor-Related Protein 1 as a Potential Therapeutic Target in Alzheimer's Disease. Pharmaceutics, 16. 10.3390/pharmaceutics16070948 PMC11279518 39065645

[49] Deane R, Du Yan S, Submamaryan RK, LaRue B, Jovanovic S, Hogg E, et al. (2003). RAGE mediates amyloid-beta peptide transport across the blood-brain barrier and accumulation in brain. Nat Med, 9:907-913. 10.1038/nm890 12808450

[50] Takeshita Y, Ransohoff RM (2012). Inflammatory cell trafficking across the blood-brain barrier: chemokine regulation and in vitro models. Immunol Rev, 248:228-239. 10.1111/j.1600-065X.2012.01127.x PMC3383666 22725965

[51] Hawkins BT, Lundeen TF, Norwood KM, Brooks HL, Egleton RD (2007). Increased blood-brain barrier permeability and altered tight junctions in experimental diabetes in the rat: contribution of hyperglycaemia and matrix metalloproteinases. Diabetologia, 50:202-211. 10.1007/s00125-006-0485-z 17143608

[52] Chehade JM, Haas MJ, Mooradian AD (2002). Diabetes-related changes in rat cerebral occludin and zonula occludens-1 (ZO-1) expression. Neurochem Res, 27:249-252. 10.1023/a:1014892706696 11958524

[53] Rom S, Heldt NA, Gajghate S, Seliga A, Reichenbach NL, Persidsky Y (2020). Hyperglycemia and advanced glycation end products disrupt BBB and promote occludin and claudin-5 protein secretion on extracellular microvesicles. Sci Rep, 10:7274. 10.1038/s41598-020-64349-x PMC7190636 32350344

[54] Liu Y, Zhang H, Wang S, Guo Y, Fang X, Zheng B, et al. (2021). Reduced pericyte and tight junction coverage in old diabetic rats are associated with hyperglycemia-induced cerebrovascular pericyte dysfunction. Am J Physiol Heart Circ Physiol, 320:H549-h562. 10.1152/ajpheart.00726.2020 PMC8082790 33306445

[55] Sajja RK, Prasad S, Cucullo L (2014). Impact of altered glycaemia on blood-brain barrier endothelium: an in vitro study using the hCMEC/D3 cell line. Fluids Barriers CNS, 11:8. 10.1186/2045-8118-11-8 PMC3985548 24708805

[56] Xu ZY, Fu SX, Zhao HC, Wang YM, Liu Y, Ma JY, et al. (2024). Dynamic changes in key factors of the blood-brain barrier in early diabetic mice. [J] Neuropathol Exp Neurol. 10.1093/jnen/nlae056 38874450

[57] Yoo DY, Yim HS, Jung HY, Nam SM, Kim JW, Choi JH, et al. (2016). Chronic type 2 diabetes reduces the integrity of the blood-brain barrier by reducing tight junction proteins in the hippocampus. J Vet Med Sci, 78:957-962. 10.1292/jvms.15-0589 PMC4937155 26876499

[58] Sajja RK, Cucullo L (2015). Altered glycaemia differentially modulates efflux transporter expression and activity in hCMEC/D3 cell line. Neurosci Lett, 598:59-65. 10.1016/j.neulet.2015.05.015 PMC4456266 25982326

[59] Rom S, Zuluaga-Ramirez V, Gajghate S, Seliga A, Winfield M, Heldt NA, et al. (2019). Hyperglycemia-Driven Neuroinflammation Compromises BBB Leading to Memory Loss in Both Diabetes Mellitus (DM) Type 1 and Type 2 Mouse Models. Mol Neurobiol, 56:1883-1896. 10.1007/s12035-018-1195-5 PMC6320739 29974394

[60] Salameh TS, Shah GN, Price TO, Hayden MR, Banks WA (2016). Blood-Brain Barrier Disruption and Neurovascular Unit Dysfunction in Diabetic Mice: Protection with the Mitochondrial Carbonic Anhydrase Inhibitor Topiramate. J Pharmacol Exp Ther, 359:452-459. 10.1124/jpet.116.237057 PMC5118649 27729477

[61] Cui X, Chopp M, Zacharek A, Ye X, Roberts C, Chen J (2011). Angiopoietin/Tie2 pathway mediates type 2 diabetes induced vascular damage after cerebral stroke. Neurobiol Dis, 43:285-292. 10.1016/j.nbd.2011.04.005 PMC3096677 21515377

[62] Li X, Yin Q, Han X, Zhang H, Wang F, Ma J, et al. (2021). Dynamic expression of vascular endothelial growth factor (VEGF) and platelet-derived growth factor receptor beta (PDGFRβ) in diabetic brain contributes to cognitive dysfunction. Brain Res Bull, 175:99-106. 10.1016/j.brainresbull.2021.07.017 34303767

[63] Liu Y, Chen D, Smith A, Ye Q, Gao Y, Zhang W (2021). Three-dimensional remodeling of functional cerebrovascular architecture and gliovascular unit in leptin receptor-deficient mice. J Cereb Blood Flow Metab, 41:1547-1562. 10.1177/0271678X211006596 PMC8221780 33818188

[64] Özkan E, Çetin-Taş Y, Şekerdağ E, Yiğit B, Shomalizadeh N, Sapancı S, et al. (2023). Hyperglycemia with or without insulin resistance triggers different structural changes in brain microcirculation and perivascular matrix. Metab Brain Dis, 38:307-321. 10.1007/s11011-022-01100-7 36305999

[65] Bouchard P, Ghitescu LD, Bendayan M (2002). Morpho-functional studies of the blood-brain barrier in streptozotocin-induced diabetic rats. Diabetologia, 45:1017-1025. 10.1007/s00125-002-0853-2 12136401

[66] Puig-Pijoan A, Jimenez-Balado J, Fernández-Lebrero A, García-Escobar G, Navalpotro-Gómez I, Contador J, et al. (2024). Risk of cognitive decline progression is associated to increased blood-brain-barrier permeability: A longitudinal study in a memory unit clinical cohort. Alzheimers Dement, 20:538-548. 10.1002/alz.13433 PMC10916969 37727082

[67] Tian X, Zhao Y, Zhu Y, Cui M (2024). Association between elevated blood-brain barrier permeability and the risk of progressive cognitive decline: A longitudinal study. Arch Gerontol Geriatr, 124:105441. 10.1016/j.archger.2024.105441 38643666

[68] Gan J, Yang X, Zhang G, Li X, Liu S, Zhang W, et al. (2023). Alzheimer's disease pathology: pathways between chronic vascular risk factors and blood-brain barrier dysfunction in a cohort of patients with different types of dementia. Front Aging Neurosci, 15:1088140. 10.3389/fnagi.2023.1088140 PMC10194826 37213537

[69] Janelidze S, Hertze J, Nägga K, Nilsson K, Nilsson C, Wennström M, et al. (2017). Increased blood-brain barrier permeability is associated with dementia and diabetes but not amyloid pathology or APOE genotype. Neurobiol Aging, 51:104-112. 10.1016/j.neurobiolaging.2016.11.017 PMC5754327 28061383

[70] Bakhtiari A, Vestergaard MB, Benedek K, Fagerlund B, Mortensen EL, Osler M, et al. (2023). Changes in hippocampal volume during a preceding 10-year period do not correlate with cognitive performance and hippocampal blood–brain barrier permeability in cognitively normal late-middle-aged men. Geroscience, 45:1161-1175. 10.1007/s11357-022-00712-2 PMC9886720 36534276

[71] Starr JM, Wardlaw J, Ferguson K, MacLullich A, Deary IJ, Marshall I (2003). Increased blood-brain barrier permeability in type II diabetes demonstrated by gadolinium magnetic resonance imaging. J Neurol Neurosurg Psychiatry, 74:70-76. 10.1136/jnnp.74.1.70 PMC1738177 12486269

[72] Acharya NK, Levin EC, Clifford PM, Han M, Tourtellotte R, Chamberlain D, et al. (2013). Diabetes and hypercholesterolemia increase blood-brain barrier permeability and brain amyloid deposition: beneficial effects of the LpPLA2 inhibitor darapladib. J Alzheimers Dis, 35:179-198. 10.3233/JAD-122254 23388174

[73] Hawkins BT, Ocheltree SM, Norwood KM, Egleton RD (2007). Decreased blood-brain barrier permeability to fluorescein in streptozotocin-treated rats. Neurosci Lett, 411:1-5. 10.1016/j.neulet.2006.09.010 PMC1785293 17110033

[74] Hosny SS, Bahaaeldin AM, Khater MS, Bekhet MM, Hebah HA, Hasanin GA (2019). Role of Inflammatory Markers in Elderly Type 2 Diabetic Patients with Mild Cognitive Impairment. Curr Diabetes Rev, 15:247-253. 10.2174/1573399814666180423113341 29683094

[75] Chen YC, Lu BZ, Shu YC, Sun YT (2021). Spatiotemporal Dynamics of Cerebral Vascular Permeability in Type 2 Diabetes-Related Cerebral Microangiopathy. Front Endocrinol (Lausanne), 12:805637. 10.3389/fendo.2021.805637 PMC8786705 35087478

[76] Wong SM, Jansen JFA, Zhang CE, Hoff EI, Staals J, van Oostenbrugge RJ, et al. (2019). Blood-brain barrier impairment and hypoperfusion are linked in cerebral small vessel disease. Neurology, 92:e1669-e1677. 10.1212/WNL.0000000000007263 30867275

[77] Hernández B, Dyer AH, Finucane C, Nipoti B, Romero-Ortuno R, Reilly R, et al. (2024). The Impact of Type 2 Diabetes on Peripheral and Cerebral Hemodynamic Responses to Active Stand. J Gerontol A Biol Sci Med Sci, 79. 10.1093/gerona/glae073 PMC11025558 38436476

[78] Cerasuolo J, Izzo A (2017). Persistent impairment in working memory following severe hyperglycemia in newly diagnosed type 2 diabetes. Endocrinol Diabetes Metab Case Rep, 2017. 10.1530/EDM-17-0101 PMC5744618 29302328

[79] Exalto LG, Biessels GJ, Karter AJ, Huang ES, Quesenberry CP Jr., Whitmer RA (2014). Severe diabetic retinal disease and dementia risk in type 2 diabetes. J Alzheimers Dis, 42 Suppl 3:S109-117. 10.3233/JAD-132570 PMC4373321 24625797

[80] Mielke MM, Evans JK, Neiberg RH, Molina-Henry DP, Marcovina SM, Johnson KC, et al. (2025). Alzheimer Disease Blood Biomarkers and Cognition Among Individuals With Diabetes and Overweight or Obesity. JAMA Netw Open, 8:e2458149. 10.1001/jamanetworkopen.2024.58149 PMC11803481 39913137

[81] Mast H, Thompson JL, Lee SH, Mohr JP, Sacco RL (1995). Hypertension and diabetes mellitus as determinants of multiple lacunar infarcts. Stroke, 26:30-33. 10.1161/01.str.26.1.30 7839393

[82] Tuttolomondo A, Pinto A, Salemi G, Di Raimondo D, Di Sciacca R, Fernandez P, et al. (2008). Diabetic and non-diabetic subjects with ischemic stroke: differences, subtype distribution and outcome. Nutr Metab Cardiovasc Dis, 18:152-157. 10.1016/j.numecd.2007.02.003 17702553

[83] Homoud B, Alhakami A, Almalki M, Shaheen M, Althuabiti A, AlKhathaami A, et al. (2020). The association of diabetes with ischemic stroke and transient ischemic attacks in a tertiary center in Saudi Arabia. Ann Saudi Med, 40:449-455. 10.5144/0256-4947.2020.449 PMC7733642 33307739

[84] Latour LL, Kang DW, Ezzeddine MA, Chalela JA, Warach S (2004). Early blood-brain barrier disruption in human focal brain ischemia. Ann Neurol, 56:468-477. 10.1002/ana.20199 15389899

[85] Giraud M, Cho TH, Nighoghossian N, Maucort-Boulch D, Deiana G, Østergaard L, et al. (2015). Early Blood Brain Barrier Changes in Acute Ischemic Stroke: A Sequential MRI Study. J Neuroimaging, 25:959-963. 10.1111/jon.12225 25702824

[86] Serlin Y, Ofer J, Ben-Arie G, Veksler R, Ifergane G, Shelef I, et al. (2019). Blood-Brain Barrier Leakage: A New Biomarker in Transient Ischemic Attacks. Stroke, 50:1266-1269. 10.1161/STROKEAHA.119.025247 31009340

[87] Yu X, Xu X, Jackson A, Sun J, Huang P, Mao Y, et al. (2016). Blood Brain Barrier Disruption in Diabetic Stroke Related to Unfavorable Outcome. Cerebrovasc Dis, 42:49-56. 10.1159/000444809 26986824

[88] Kawamura T, Umemura T, Kanai A, Nagashima M, Nakamura N, Uno T, et al. (2006). Soluble adhesion molecules and C-reactive protein in the progression of silent cerebral infarction in patients with type 2 diabetes mellitus. Metabolism, 55:461-466. 10.1016/j.metabol.2005.10.007 16546476

[89] Kanai A, Kawamura T, Umemura T, Nagashima M, Nakamura N, Nakayama M, et al. (2008). Association between future events of brain infarction and soluble levels of intercellular adhesion molecule-1 and C-reactive protein in patients with type 2 diabetes mellitus. Diabetes Res Clin Pract, 82:157-164. 10.1016/j.diabres.2008.07.006 18692933

[90] Otero-Ortega L, Gutiérrez-Fernández M, Gutiérrez-Zúñiga R, Madero-Jarabo R, Alonso de Leciñana M, Laso-García F, et al. (2019). The effect of post-stroke hyperglycaemia on the levels of brain damage and repair-related circulating biomarkers: the Glycaemia in Acute Stroke Study II. Eur J Neurol, 26:1439-1446. 10.1111/ene.14010 31141256

[91] Serlin Y, Shafat T, Levy J, Winter A, Shneck M, Knyazer B, et al. (2016). Angiographic evidence of proliferative retinopathy predicts neuropsychiatric morbidity in diabetic patients. Psychoneuroendocrinology, 67:163-170. 10.1016/j.psyneuen.2016.02.009 26907995

[92] Hom J, Dankbaar JW, Soares BP, Schneider T, Cheng SC, Bredno J, et al. (2011). Blood-brain barrier permeability assessed by perfusion CT predicts symptomatic hemorrhagic transformation and malignant edema in acute ischemic stroke. AJNR Am J Neuroradiol, 32:41-48. 10.3174/ajnr.A2244 PMC7964964 20947643

[93] Duarte-Silva E, de Melo MG, Maes M, Filho A, Macedo D, Peixoto CA (2021). Shared metabolic and neuroimmune mechanisms underlying Type 2 Diabetes Mellitus and Major Depressive Disorder. Prog Neuropsychopharmacol Biol Psychiatry, 111:110351. 10.1016/j.pnpbp.2021.110351 34000290

[94] Ogunsakin RE, Olugbara OO, Moyo S, Israel C (2021). Meta-analysis of studies on depression prevalence among diabetes mellitus patients in Africa. Heliyon, 7:e07085. 10.1016/j.heliyon.2021.e07085 PMC8165422 34095580

[95] van Dooren FE, Nefs G, Schram MT, Verhey FR, Denollet J, Pouwer F (2013). Depression and risk of mortality in people with diabetes mellitus: a systematic review and meta-analysis. PLoS One, 8:e57058. 10.1371/journal.pone.0057058 PMC3589463 23472075

[96] Gudmundsson P, Skoog I, Waern M, Blennow K, Pálsson S, Rosengren L, et al. (2007). The relationship between cerebrospinal fluid biomarkers and depression in elderly women. Am J Geriatr Psychiatry, 15:832-838. 10.1097/JGP.0b013e3180547091 17911361

[97] Niklasson F, Agren H (1984). Brain energy metabolism and blood-brain barrier permeability in depressive patients: analyses of creatine, creatinine, urate, and albumin in CSF and blood. Biol Psychiatry, 19:1183-1206. 6498242

[98] Zachrisson OC, Balldin J, Ekman R, Naesh O, Rosengren L, Agren H, et al. (2000). No evident neuronal damage after electroconvulsive therapy. Psychiatry Res, 96:157-165. 10.1016/s0165-1781(00)00202-x 11063788

[99] Schroeter ML, Abdul-Khaliq H, Krebs M, Diefenbacher A, Blasig IE (2008). Serum markers support disease-specific glial pathology in major depression. J Affect Disord, 111:271-280. 10.1016/j.jad.2008.03.005 18430474

[100] Herder C, Fürstos JF, Nowotny B, Begun A, Strassburger K, Müssig K, et al. (2017). Associations between inflammation-related biomarkers and depressive symptoms in individuals with recently diagnosed type 1 and type 2 diabetes. Brain Behav Immun, 61:137-145. 10.1016/j.bbi.2016.12.025 28041985

[101] Laake J-PS, Stahl D, Amiel SA, Petrak F, Sherwood RA, Pickup JC, et al. (2014). The Association Between Depressive Symptoms and Systemic Inflammation in People With Type 2 Diabetes: Findings From the South London Diabetes Study. Diabetes Care, 37:2186-2192. 10.2337/dc13-2522 24842983

[102] Liu Y, Ho RC, Mak A (2012). Interleukin (IL)-6, tumour necrosis factor alpha (TNF-α) and soluble interleukin-2 receptors (sIL-2R) are elevated in patients with major depressive disorder: a meta-analysis and meta-regression. J Affect Disord, 139:230-239. 10.1016/j.jad.2011.08.003 21872339

[103] Fujii T, Ota M, Hori H, Sasayama D, Hattori K, Teraishi T, et al. (2012). Association between the functional polymorphism (C3435T) of the gene encoding P-glycoprotein (ABCB1) and major depressive disorder in the Japanese population. J Psychiatr Res, 46:555-559. 10.1016/j.jpsychires.2012.01.012 22306099

[104] Kamintsky L, Cairns KA, Veksler R, Bowen C, Beyea SD, Friedman A, et al. (2020). Blood-brain barrier imaging as a potential biomarker for bipolar disorder progression. Neuroimage Clin, 26:102049. 10.1016/j.nicl.2019.102049 PMC7229352 31718955

[105] Calkin C, McClelland C, Cairns K, Kamintsky L, Friedman A (2021). Insulin Resistance and Blood-Brain Barrier Dysfunction Underlie Neuroprogression in Bipolar Disorder. Front Psychiatry, 12:636174. 10.3389/fpsyt.2021.636174 PMC8185298 34113269

[106] Nouwen A, Adriaanse MC, van Dam K, Iversen MM, Viechtbauer W, Peyrot M, et al. (2019). Longitudinal associations between depression and diabetes complications: a systematic review and meta-analysis. Diabet Med, 36:1562-1572. 10.1111/dme.14054 31215077

[107] Nguyen TT, Wong TY, Islam FM, Hubbard L, Ajilore O, Haroon E, et al. (2010). Evidence of early retinal microvascular changes in patients with type 2 diabetes and depression. Psychosom Med, 72:535-538. 10.1097/PSY.0b013e3181da90f4 20368470

[108] Lo JW, Crawford JD, Samaras K, Desmond DW, Köhler S, Staals J, et al. (2020). Association of Prediabetes and Type 2 Diabetes With Cognitive Function After Stroke: A STROKOG Collaboration Study. Stroke, 51:1640-1646. 10.1161/STROKEAHA.119.028428 32404039

[109] Cukierman-Yaffe T, McClure LA, Risoli T, Bosch J, Sharma M, Gerstein HC, et al. (2021). The Relationship Between Glucose Control and Cognitive Function in People With Diabetes After a Lacunar Stroke. J Clin Endocrinol Metab, 106:e1521-e1528. 10.1210/clinem/dgab022 PMC7993572 33481011

[110] Huang YC, Cuevas HE, Zuñiga JA, García AA (2021). Predictors of Subjective Cognitive Decline Among People With Diabetes: Data From the Behavioral Risk Factor Surveillance System. Sci Diabetes Self Manag Care, 47:207-215. 10.1177/26350106211001761 34000913

[111] Yang S, Boudier-Revéret M, Kwon S, Lee MY, Chang MC (2021). Effect of Diabetes on Post-stroke Recovery: A Systematic Narrative Review. Front Neurol, 12:747878. 10.3389/fneur.2021.747878 PMC8712454 34970205

[112] Ma ZY, Wu YY, Cui HY, Yao GY, Bian H (2022). Factors Influencing Post-Stroke Cognitive Impairment in Patients with Type 2 Diabetes Mellitus. Clin Interv Aging, 17:653-664. 10.2147/CIA.S355242 PMC9063799 35520948

[113] Zhong P, Tan S, Zhu Z, Zhang J, Chen S, Huang W, et al. (2023). Brain and Cognition Signature Fingerprinting Vascular Health in Diabetic Individuals: An International Multi-Cohort Study. Am J Geriatr Psychiatry, 31:570-582. 10.1016/j.jagp.2023.04.010 37230837

[114] Jokisch M, Schramm S, Weimar C, Moebus S, Gronewold J, Dragano N, et al. (2022). Fluctuation of depressive symptoms in cognitively unimpaired participants and the risk of mild cognitive impairment 5 years later: Results of the Heinz Nixdorf Recall study. Front Behav Neurosci, 16:988621. 10.3389/fnbeh.2022.988621 PMC9640513 36386784

[115] Kim DW, Moon Y, Gee Noh H, Choi JW, Oh J (2011). Blood-brain barrier disruption is involved in seizure and hemianopsia in nonketotic hyperglycemia. Neurologist, 17:164-166. 10.1097/NRL.0b013e3182173528 21532388

[116] Navasardyan I, Yeganyan S, Nguyen H, Vaghashia P, Subbian S, Venketaraman V (2023). Role of Oxidative Stress in Tuberculosis Meningitis Infection in Diabetics. Biomedicines, 11. 10.3390/biomedicines11092568 PMC10526095 37761009

[117] Elabi OF, Cunha J, Gaceb A, Fex M, Paul G (2021). High-fat diet-induced diabetes leads to vascular alterations, pericyte reduction, and perivascular depletion of microglia in a 6-OHDA toxin model of Parkinson disease. J Neuroinflammation, 18:175. 10.1186/s12974-021-02218-8 PMC8353816 34376193

[118] Chen A, Duan Y, Zhou S, Du F, Peng H, Zeng D, et al. (2025). Mesenchymal Stem Cells Restore Endothelial Integrity and Alleviate Emotional Impairments in a Diabetic Mouse Model via Inhibition of MMP-9 Activity. Int J Mol Sci, 26. 10.3390/ijms26073355 PMC11989596 40244194

[119] Mersha AG, Tollosa DN, Bagade T, Eftekhari P (2022). A bidirectional relationship between diabetes mellitus and anxiety: A systematic review and meta-analysis. J Psychosom Res, 162:110991. 10.1016/j.jpsychores.2022.110991 36081182

[120] Dion-Albert L, Cadoret A, Doney E, Kaufmann FN, Dudek KA, Daigle B, et al. (2022). Vascular and blood-brain barrier-related changes underlie stress responses and resilience in female mice and depression in human tissue. Nat Commun, 13:164. 10.1038/s41467-021-27604-x PMC8748803 35013188

[121] Sumbul-Sekerci B, Pasin O, Balkan E, Sekerci A (2025). The Role of Inflammation, Oxidative Stress, Neuronal Damage, and Endothelial Dysfunction in the Neuropathology of Cognitive Complications in Diabetes: A Moderation and Mediation Analysis. Brain Behav, 15:e70225. 10.1002/brb3.70225 PMC11743999 39829127

[122] Niu J, Ran Y, Chen R, Zhang Y, Zhang Y, Yang Q, et al. (2025). Evaluation of Middle Cerebral Artery Culprit Plaque Inflammation in Ischemic Stroke Using CAIPIRINHA-Dixon-TWIST Dynamic Contrast-Enhanced Magnetic Resonance Imaging. J Magn Reson Imaging, 61:2011-2020. 10.1002/jmri.29576 39258494

[123] Gorska-Ciebiada M, Saryusz-Wolska M, Borkowska A, Ciebiada M, Loba J (2015). Serum Soluble Adhesion Molecules and Markers of Systemic Inflammation in Elderly Diabetic Patients with Mild Cognitive Impairment and Depressive Symptoms. Biomed Res Int, 2015:826180. 10.1155/2015/826180 PMC4488515 26167502

[124] Novak V, Zhao P, Manor B, Sejdic E, Alsop D, Abduljalil A, et al. (2011). Adhesion molecules, altered vasoreactivity, and brain atrophy in type 2 diabetes. Diabetes Care, 34:2438-2441. 10.2337/dc11-0969 PMC3198286 21926285

[125] Jack CR Jr., Knopman DS, Jagust WJ, Petersen RC, Weiner MW, Aisen PS, et al. (2013). Tracking pathophysiological processes in Alzheimer's disease: an updated hypothetical model of dynamic biomarkers. Lancet Neurol, 12:207-216. 10.1016/S1474-4422(12)70291-0 PMC3622225 23332364

[126] Yan J, Zhang Z, Shi H (2012). HIF-1 is involved in high glucose-induced paracellular permeability of brain endothelial cells. Cell Mol Life Sci, 69:115-128. 10.1007/s00018-011-0731-5 PMC11115066 21617913

[127] Qiao J, Lawson CM, Rentrup KFG, Kulkarni P, Ferris CF (2020). Evaluating blood-brain barrier permeability in a rat model of type 2 diabetes. J Transl Med, 18:256. 10.1186/s12967-020-02428-3 PMC7313122 32580725

[128] Huber JD, VanGilder RL, Houser KA (2006). Streptozotocin-induced diabetes progressively increases blood-brain barrier permeability in specific brain regions in rats. Am J Physiol Heart Circ Physiol, 291:H2660-2668. 10.1152/ajpheart.00489.2006 16951046

[129] Liu H, Xu X, Yang Z, Deng Y, Liu X, Xie L (2006). Impaired function and expression of P-glycoprotein in blood-brain barrier of streptozotocin-induced diabetic rats. Brain Res, 1123:245-252. 10.1016/j.brainres.2006.09.061 17074306

[130] Ogata S, Ito S, Masuda T, Ohtsuki S (2019). Changes of Blood-Brain Barrier and Brain Parenchymal Protein Expression Levels of Mice under Different Insulin-Resistance Conditions Induced by High-Fat Diet. Pharm Res, 36:141. 10.1007/s11095-019-2674-8 31367840

[131] Jakobsen J, Knudsen GM, Juhler M (1987). Cation permeability of the blood-brain barrier in streptozotocin-diabetic rats. Diabetologia, 30:409-413. 10.1007/BF00292543 3678661

[132] Liu X, Sui B, Sun J (2017). Blood-brain barrier dysfunction induced by silica NPs in vitro and in vivo: Involvement of oxidative stress and Rho-kinase/JNK signaling pathways. Biomaterials, 121:64-82. 10.1016/j.biomaterials.2017.01.006 28081460

[133] Yu T, Robotham JL, Yoon Y (2006). Increased production of reactive oxygen species in hyperglycemic conditions requires dynamic change of mitochondrial morphology. Proc Natl Acad Sci U S A, 103:2653-2658. 10.1073/pnas.0511154103 PMC1413838 16477035

[134] Du XL, Edelstein D, Rossetti L, Fantus IG, Goldberg H, Ziyadeh F, et al. (2000). Hyperglycemia-induced mitochondrial superoxide overproduction activates the hexosamine pathway and induces plasminogen activator inhibitor-1 expression by increasing Sp1 glycosylation. Proc Natl Acad Sci U S A, 97:12222-12226. 10.1073/pnas.97.22.12222 PMC17322 11050244

[135] Allen CL, Bayraktutan U (2009). Antioxidants attenuate hyperglycaemia-mediated brain endothelial cell dysfunction and blood-brain barrier hyperpermeability. Diabetes Obes Metab, 11:480-490. 10.1111/j.1463-1326.2008.00987.x 19236439

[136] Anasooya Shaji C, Robinson BD, Yeager A, Beeram MR, Davis ML, Isbell CL, et al. (2019). The Tri-phasic Role of Hydrogen Peroxide in Blood-Brain Barrier Endothelial cells. Sci Rep, 9:133. 10.1038/s41598-018-36769-3 PMC6333800 30644421

[137] Schreibelt G, Kooij G, Reijerkerk A, van Doorn R, Gringhuis SI, van der Pol S, et al. (2007). Reactive oxygen species alter brain endothelial tight junction dynamics via RhoA, PI3 kinase, and PKB signaling. Faseb j, 21:3666-3676. 10.1096/fj.07-8329com 17586731

[138] Price TO, Eranki V, Banks WA, Ercal N, Shah GN (2012). Topiramate treatment protects blood-brain barrier pericytes from hyperglycemia-induced oxidative damage in diabetic mice. Endocrinology, 153:362-372. 10.1210/en.2011-1638 PMC3249670 22109883

[139] Shah GN, Morofuji Y, Banks WA, Price TO (2013). High glucose-induced mitochondrial respiration and reactive oxygen species in mouse cerebral pericytes is reversed by pharmacological inhibition of mitochondrial carbonic anhydrases: Implications for cerebral microvascular disease in diabetes. Biochem Biophys Res Commun, 440:354-358. 10.1016/j.bbrc.2013.09.086 PMC3875343 24076121

[140] Salameh TS, Mortell WG, Logsdon AF, Butterfield DA, Banks WA (2019). Disruption of the hippocampal and hypothalamic blood-brain barrier in a diet-induced obese model of type II diabetes: prevention and treatment by the mitochondrial carbonic anhydrase inhibitor, topiramate. Fluids Barriers CNS, 16:1. 10.1186/s12987-018-0121-6 PMC6323732 30616618

[141] Arcambal A, Taïlé J, Rondeau P, Viranaïcken W, Meilhac O, Gonthier MP (2019). Hyperglycemia modulates redox, inflammatory and vasoactive markers through specific signaling pathways in cerebral endothelial cells: Insights on insulin protective action. Free Radic Biol Med, 130:59-70. 10.1016/j.freeradbiomed.2018.10.430 30359759

[142] Kusaka I, Kusaka G, Zhou C, Ishikawa M, Nanda A, Granger DN, et al. (2004). Role of AT1 receptors and NAD(P)H oxidase in diabetes-aggravated ischemic brain injury. Am J Physiol Heart Circ Physiol, 286:H2442-2451. 10.1152/ajpheart.01169.2003 15148062

[143] Sheikh MH, Errede M, d'Amati A, Khan NQ, Fanti S, Loiola RA, et al. (2022). Impact of metabolic disorders on the structural, functional, and immunological integrity of the blood-brain barrier: Therapeutic avenues. Faseb j, 36:e22107. 10.1096/fj.202101297R 34939700

[144] Vittal Rao H, Bihaqi SW, Iannucci J, Sen A, Grammas P (2021). Thrombin Signaling Contributes to High Glucose-Induced Injury of Human Brain Microvascular Endothelial Cells. J Alzheimers Dis, 79:211-224. 10.3233/JAD-200658 33252072

[145] Taïlé J, Patché J, Veeren B, Gonthier MP (2021). Hyperglycemic Condition Causes Pro-Inflammatory and Permeability Alterations Associated with Monocyte Recruitment and Deregulated NFκB/PPARγ Pathways on Cerebral Endothelial Cells: Evidence for Polyphenols Uptake and Protective Effect. Int J Mol Sci, 22. 10.3390/ijms22031385 PMC7866545 33573189

[146] Geng J, Wang L, Zhang L, Qin C, Song Y, Ma Y, et al. (2018). Blood-Brain Barrier Disruption Induced Cognitive Impairment Is Associated With Increase of Inflammatory Cytokine. Front Aging Neurosci, 10:129. 10.3389/fnagi.2018.00129 PMC5949351 29867440

[147] Close TE, Cepinskas G, Omatsu T, Rose KL, Summers K, Patterson EK, et al. (2013). Diabetic ketoacidosis elicits systemic inflammation associated with cerebrovascular endothelial cell dysfunction. Microcirculation, 20:534-543. 10.1111/micc.12053 23441883

[148] Kim H, Leng K, Park J, Sorets AG, Kim S, Shostak A, et al. (2022). Reactive astrocytes transduce inflammation in a blood-brain barrier model through a TNF-STAT3 signaling axis and secretion of alpha 1-antichymotrypsin. Nat Commun, 13:6581. 10.1038/s41467-022-34412-4 PMC9630454 36323693

[149] Wei C, Jiang W, Wang R, Zhong H, He H, Gao X, et al. (2024). Brain endothelial GSDMD activation mediates inflammatory BBB breakdown. Nature, 629:893-900. 10.1038/s41586-024-07314-2 38632402

[150] Hoshi Y, Uchida Y, Tachikawa M, Ohtsuki S, Terasaki T (2017). Actin filament-associated protein 1 (AFAP-1) is a key mediator in inflammatory signaling-induced rapid attenuation of intrinsic P-gp function in human brain capillary endothelial cells. J Neurochem, 141:247-262. 10.1111/jnc.13960 28112407

[151] Pugazhenthi S, Qin L, Reddy PH (2017). Common neurodegenerative pathways in obesity, diabetes, and Alzheimer's disease. Biochim Biophys Acta Mol Basis Dis, 1863:1037-1045. 10.1016/j.bbadis.2016.04.017 PMC5344771 27156888

[152] Beeri MS, Uribarri J, Cai W, Buchman AS, Haroutunian V (2020). Human Brain and Serum Advanced Glycation End Products are Highly Correlated: Preliminary Results of Their Role in Alzheimer Disease and Type 2 Diabetes. Endocr Pract, 26:576-577. 10.4158/1934-2403-26.5.576 PMC8254854 32396777

[153] Li W, Maloney RE, Aw TY (2015). High glucose, glucose fluctuation and carbonyl stress enhance brain microvascular endothelial barrier dysfunction: Implications for diabetic cerebral microvasculature. Redox Biol, 5:80-90. 10.1016/j.redox.2015.03.005 PMC4398791 25867911

[154] Takeshita T, Nakagawa S, Tatsumi R, So G, Hayashi K, Tanaka K, et al. (2014). Cilostazol attenuates ischemia-reperfusion-induced blood-brain barrier dysfunction enhanced by advanced glycation endproducts via transforming growth factor-β1 signaling. Mol Cell Neurosci, 60:1-9. 10.1016/j.mcn.2014.01.006 24472843

[155] Shimizu F, Sano Y, Tominaga O, Maeda T, Abe MA, Kanda T (2013). Advanced glycation end-products disrupt the blood-brain barrier by stimulating the release of transforming growth factor-β by pericytes and vascular endothelial growth factor and matrix metalloproteinase-2 by endothelial cells in vitro. Neurobiol Aging, 34:1902-1912. 10.1016/j.neurobiolaging.2013.01.012 23428182

[156] Zhang F, Banker G, Liu X, Suwanabol PA, Lengfeld J, Yamanouchi D, et al. (2011). The novel function of advanced glycation end products in regulation of MMP-9 production. J Surg Res, 171:871-876. 10.1016/j.jss.2010.04.027 PMC3623272 20638679

[157] Liu N, Liu C, Yang Y, Ma G, Wei G, Liu S, et al. (2021). Xiao-Xu-Ming decoction prevented hemorrhagic transformation induced by acute hyperglycemia through inhibiting AGE-RAGE-mediated neuroinflammation. Pharmacol Res, 169:105650. 10.1016/j.phrs.2021.105650 33964468

[158] Yao D, Brownlee M (2010). Hyperglycemia-induced reactive oxygen species increase expression of the receptor for advanced glycation end products (RAGE) and RAGE ligands. Diabetes, 59:249-255. 10.2337/db09-0801 PMC2797929 19833897

[159] Skrha J Jr., Kalousová M, Svarcová J, Muravská A, Kvasnička J, Landová L, et al. (2012). Relationship of soluble RAGE and RAGE ligands HMGB1 and EN-RAGE to endothelial dysfunction in type 1 and type 2 diabetes mellitus. Exp Clin Endocrinol Diabetes, 120:277-281. 10.1055/s-0031-1283161 22549347

[160] Hu J, Liu B, Zhao Q, Jin P, Hua F, Zhang Z, et al. (2016). Bone marrow stromal cells inhibits HMGB1-mediated inflammation after stroke in type 2 diabetic rats. Neuroscience, 324:11-19. 10.1016/j.neuroscience.2016.02.058 26946264

[161] Festoff BW, Sajja RK, van Dreden P, Cucullo L (2016). HMGB1 and thrombin mediate the blood-brain barrier dysfunction acting as biomarkers of neuroinflammation and progression to neurodegeneration in Alzheimer's disease. J Neuroinflammation, 13:194. 10.1186/s12974-016-0670-z PMC4995775 27553758

[162] Mondal A, Bose D, Saha P, Sarkar S, Seth R, Kimono D, et al. (2020). Lipocalin 2 induces neuroinflammation and blood-brain barrier dysfunction through liver-brain axis in murine model of nonalcoholic steatohepatitis. J Neuroinflammation, 17:201. 10.1186/s12974-020-01876-4 PMC7335438 32622362

[163] Zhou H, Jin C, Cui L, Xing H, Liu J, Liao W, et al. (2018). HMGB1 contributes to the irradiation-induced endothelial barrier injury through receptor for advanced glycation endproducts (RAGE). J Cell Physiol, 233:6714-6721. 10.1002/jcp.26341 PMC13482486 29215715

[164] Wang Y, Wang Q, Luo D, Zhao P, Zhong SS, Dai B, et al. (2023). Electroacupuncture Improves Blood-Brain Barrier and Hippocampal Neuroinflammation in SAMP8 Mice by Inhibiting HMGB1/TLR4 and RAGE/NADPH Signaling Pathways. Chin J Integr Med, 29:448-458. 10.1007/s11655-023-3592-5 36609953

[165] Liao YC, Wang JW, Guo C, Bai M, Ran Z, Wen LM, et al. (2023). Cistanche tubulosa alleviates ischemic stroke-induced blood-brain barrier damage by modulating microglia-mediated neuroinflammation. J Ethnopharmacol, 309:116269. 10.1016/j.jep.2023.116269 36863639

[166] Binsaleh AY, Ali LS, Bahaa MM, Elmasry TA, Negm WA, Hamouda AO, et al. (2024). Correlation between depression scores and serum NF-ĸB/NLRP3 axis, biotinidase, and HMGB1 after treatment with isotretinoin in patients with acne vulgaris. [J] Cosmet Dermatol. 10.1111/jocd.16433 PMC13648887 38923374

[167] Zhu R, Zhao X, Wu H, Zeng X, Wei J, Chen T (2024). Psychobiotics Lactiplantibacillus plantarum JYLP-326: Antidepressant-like effects on CUMS-induced depressed mouse model and alleviation of gut microbiota dysbiosis. J Affect Disord, 354:752-764. 10.1016/j.jad.2024.03.136 38537753

[168] Roh HT, So WY (2017). The effects of aerobic exercise training on oxidant-antioxidant balance, neurotrophic factor levels, and blood-brain barrier function in obese and non-obese men. J Sport Health Sci, 6:447-453. 10.1016/j.jshs.2016.07.006 PMC6189263 30356625

[169] de Senna PN, Xavier LL, Bagatini PB, Saur L, Galland F, Zanotto C, et al. (2015). Physical training improves non-spatial memory, locomotor skills and the blood brain barrier in diabetic rats. Brain Res, 1618:75-82. 10.1016/j.brainres.2015.05.026 26032744

[170] Dong J, Zhao J, Lin Y, Liang H, He X, Zheng X, et al. (2018). Exercise improves recognition memory and synaptic plasticity in the prefrontal cortex for rats modelling vascular dementia. Neurol Res, 40:68-77. 10.1080/01616412.2017.1398389 29126372

[171] Liu L, Tang J, Liang X, Li Y, Zhu P, Zhou M, et al. (2024). Running exercise alleviates hippocampal neuroinflammation and shifts the balance of microglial M1/M2 polarization through adiponectin/AdipoR1 pathway activation in mice exposed to chronic unpredictable stress. Mol Psychiatry. 10.1038/s41380-024-02464-1 38361125

[172] Lu D, Qu C, Fang M, Zhang J (2024). Exercise rescues cognitive impairment through inhibiting the fibrinogen neuroinflammative pathway in diabetes. Metab Brain Dis, 40:2. 10.1007/s11011-024-01455-z 39535634

[173] Zhu A, Lin Y, Hu X, Lin Z, Lin Y, Xie Q, et al. (2022). Treadmill exercise decreases cerebral edema in rats with local cerebral infarction by modulating AQP4 polar expression through the caveolin-1/TRPV4 signaling pathway. Brain Res Bull, 188:155-168. 10.1016/j.brainresbull.2022.08.003 35961528

[174] Zhou J, Yin S, Du L, Xue X, He Q, Zhao N, et al. (2024). Independent and Combined Associations of Physical Activity in Different Domains and Inflammatory Diet with Type 2 Diabetes: A Population-Based Cohort Study. Nutrients, 17. 10.3390/nu17010047 PMC11723060 39796481

[175] Tan J, Liu N, Sun P, Tang Y, Qin W (2022). A Proinflammatory Diet May Increase Mortality Risk in Patients with Diabetes Mellitus. Nutrients, 14. 10.3390/nu14102011 PMC9145817 35631151

[176] Hodge AM, Karim MN, Hébert JR, Shivappa N, de Courten B (2021). Association between Diet Quality Indices and Incidence of Type 2 Diabetes in the Melbourne Collaborative Cohort Study. Nutrients, 13. 10.3390/nu13114162 PMC8622769 34836416

[177] Esposito K, Maiorino MI, Ciotola M, Di Palo C, Scognamiglio P, Gicchino M, et al. (2009). Effects of a Mediterranean-style diet on the need for antihyperglycemic drug therapy in patients with newly diagnosed type 2 diabetes: a randomized trial. Ann Intern Med, 151:306-314. 10.7326/0003-4819-151-5-200909010-00004 19721018

[178] Jonasson L, Guldbrand H, Lundberg AK, Nystrom FH (2014). Advice to follow a low-carbohydrate diet has a favourable impact on low-grade inflammation in type 2 diabetes compared with advice to follow a low-fat diet. Ann Med, 46:182-187. 10.3109/07853890.2014.894286 PMC4025600 24779961

[179] Lee J, An HS, Shin HJ, Jang HM, Im CO, Jeong Y, et al. (2024). Intermittent Fasting Reduces Neuroinflammation and Cognitive Impairment in High-Fat Diet-Fed Mice by Downregulating Lipocalin-2 and Galectin-3. Nutrients, 16. 10.3390/nu16010159 PMC10780385 38201988

[180] Sun Z, Liu H, Yan M, Zeng H, Hu Y, Tian X, et al. (2024). The effect of multi-component exercise on cognition function in patients with diabetes: A systematic review and meta-analysis. PLoS One, 19:e0304795. 10.1371/journal.pone.0304795 PMC11189216 38900771

[181] Garza MC, Pérez-Calahorra S, Rodrigo-Carbó C, Sánchez-Calavera MA, Jarauta E, Mateo-Gallego R, et al. (2024). Effect of Aromatic Herbs and Spices Present in the Mediterranean Diet on the Glycemic Profile in Type 2 Diabetes Subjects: A Systematic Review and Meta-Analysis. Nutrients, 16. 10.3390/nu16060756 PMC10975382 38542668

[182] Aridi YS, Walker JL, Wright ORL (2017). The Association between the Mediterranean Dietary Pattern and Cognitive Health: A Systematic Review. Nutrients, 9. 10.3390/nu9070674 PMC5537789 28657600

[183] Barbaresko J, Lellmann AW, Schmidt A, Lehmann A, Amini AM, Egert S, et al. (2020). Dietary Factors and Neurodegenerative Disorders: An Umbrella Review of Meta-Analyses of Prospective Studies. Adv Nutr, 11:1161-1173. 10.1093/advances/nmaa053 PMC7490166 32427314

[184] Zhao M, Dong Y, Chen L, Shen H (2024). Influencing factors of stroke in patients with type 2 diabetes: A systematic review and meta-analysis. PLoS One, 19:e0305954. 10.1371/journal.pone.0305954 PMC11196000 38913694

[185] Kahleova H, Salas-Salvadó J, Rahelić D, Kendall CW, Rembert E, Sievenpiper JL (2019). Dietary Patterns and Cardiometabolic Outcomes in Diabetes: A Summary of Systematic Reviews and Meta-Analyses. Nutrients, 11. 10.3390/nu11092209 PMC6770579 31540227

[186] Arsh A, Afaq S, Carswell C, Bhatti MM, Ullah I, Siddiqi N (2023). Effectiveness of physical activity in managing co-morbid depression in adults with type 2 diabetes mellitus: A systematic review and meta-analysis. J Affect Disord, 329:448-459. 10.1016/j.jad.2023.02.122 36868385

[187] Paris T, Daly RM, Abbott G, Sood S, Freer CL, Ryan MC, et al. (2024). Diet Overall and Hypocaloric Diets Are Associated With Improvements in Depression but Not Anxiety in People With Metabolic Conditions: A Systematic Review and Meta-Analysis. Adv Nutr, 15:100169. 10.1016/j.advnut.2024.100169 PMC10847486 38184198

[188] Li Z, Lin C, Cai X, Lv F, Yang W, Ji L (2024). Anti-diabetic agents and the risks of dementia in patients with type 2 diabetes: a systematic review and network meta-analysis of observational studies and randomized controlled trials. Alzheimers Res Ther, 16:272. 10.1186/s13195-024-01645-y PMC11668108 39716328

[189] Paridari P, Jabermoradi S, Gholamzadeh R, Vazifekhah S, Vazirizadeh-Mahabadi M, Roshdi Dizaji S, et al. (2023). Can metformin use reduce the risk of stroke in diabetic patients? A systematic review and meta-analysis. Diabetes Metab Syndr, 17:102721. 10.1016/j.dsx.2023.102721 36791633

[190] Moulton CD, Hopkins CWP, Ismail K, Stahl D (2018). Repositioning of diabetes treatments for depressive symptoms: A systematic review and meta-analysis of clinical trials. Psychoneuroendocrinology, 94:91-103. 10.1016/j.psyneuen.2018.05.010 29775878

[191] Hui JMH, Zhou J, Lee TTL, Hui K, Chou OHI, Lee YHA, et al. (2024). Metformin use is associated with lower risks of dementia, anxiety and depression: The Hong Kong diabetes study. Asian J Psychiatr, 97:104086. 10.1016/j.ajp.2024.104086 38796398

[192] Liu Y, Tang G, Li Y, Wang Y, Chen X, Gu X, et al. (2014). Metformin attenuates blood-brain barrier disruption in mice following middle cerebral artery occlusion. J Neuroinflammation, 11:177. 10.1186/s12974-014-0177-4 PMC4201919 25315906

[193] Han X, Wang B, Sun Y, Huang J, Wang X, Ma W, et al. (2018). Metformin Modulates High Glucose-Incubated Human Umbilical Vein Endothelial Cells Proliferation and Apoptosis Through AMPK/CREB/BDNF Pathway. Front Pharmacol, 9:1266. 10.3389/fphar.2018.01266 PMC6232387 30459620

[194] Wu Y, Chen K, Li L, Hao Z, Wang T, Liu Y, et al. (2022). Plin2-mediated lipid droplet mobilization accelerates exit from pluripotency by lipidomic remodeling and histone acetylation. Cell Death Differ, 29:2316-2331. 10.1038/s41418-022-01018-8 PMC9613632 35614132

[195] Mone P, Gambardella J, Pansini A, de Donato A, Martinelli G, Boccalone E, et al. (2021). Cognitive Impairment in Frail Hypertensive Elderly Patients: Role of Hyperglycemia. Cells, 10. 10.3390/cells10082115 PMC8391431 34440883

[196] Prasad S, Sajja RK, Kaisar MA, Park JH, Villalba H, Liles T, et al. (2017). Role of Nrf2 and protective effects of Metformin against tobacco smoke-induced cerebrovascular toxicity. Redox Biol, 12:58-69. 10.1016/j.redox.2017.02.007 PMC5312505 28212524

[197] Fakih W, Mroueh A, Salah H, Eid AH, Obeid M, Kobeissy F, et al. (2020). Dysfunctional cerebrovascular tone contributes to cognitive impairment in a non-obese rat model of prediabetic challenge: Role of suppression of autophagy and modulation by anti-diabetic drugs. Biochem Pharmacol, 178:114041. 10.1016/j.bcp.2020.114041 32439335

[198] Liu C, Zhang D, Lu Z, Man J, Zhang Z, Fu X, et al. (2022). Metformin protects against pericyte apoptosis and promotes neurogenesis through suppressing JNK p38 MAPK signalling activation in ischemia/reperfusion injury. Neurosci Lett, 783:136708. 10.1016/j.neulet.2022.136708 35660649

[199] Chen F, Dong RR, Zhong KL, Ghosh A, Tang SS, Long Y, et al. (2016). Antidiabetic drugs restore abnormal transport of amyloid-β across the blood-brain barrier and memory impairment in db/db mice. Neuropharmacology, 101:123-136. 10.1016/j.neuropharm.2015.07.023 26211973

[200] Fukuda S, Nakagawa S, Tatsumi R, Morofuji Y, Takeshita T, Hayashi K, et al. (2016). Glucagon-Like Peptide-1 Strengthens the Barrier Integrity in Primary Cultures of Rat Brain Endothelial Cells Under Basal and Hyperglycemia Conditions. J Mol Neurosci, 59:211-219. 10.1007/s12031-015-0696-1 26659380

[201] Canário NS, Crisóstomo J, Moreno C, Duarte JV, Duarte IC, Ribeiro MJ, et al. (2024). Functional reorganization of memory processing in the hippocampus is associated with neuroprotector GLP-1 levels in type 2 diabetes. Heliyon, 10:e27412. 10.1016/j.heliyon.2024.e27412 PMC10950584 38509913

[202] Hunter K, Hölscher C (2012). Drugs developed to treat diabetes, liraglutide and lixisenatide, cross the blood brain barrier and enhance neurogenesis. BMC Neurosci, 13:33. 10.1186/1471-2202-13-33 PMC3352246 22443187

[203] Zanotto C, Simão F, Gasparin MS, Biasibetti R, Tortorelli LS, Nardin P, et al. (2017). Exendin-4 Reverses Biochemical and Functional Alterations in the Blood-Brain and Blood-CSF Barriers in Diabetic Rats. Mol Neurobiol, 54:2154-2166. 10.1007/s12035-016-9798-1 26927659

[204] Xie Y, Wang Y, Ding H, Guo M, Wang X, Dong Q, et al. (2019). Highly glycosylated CD147 promotes hemorrhagic transformation after rt-PA treatment in diabetes: a novel therapeutic target? J Neuroinflammation, 16:72. 10.1186/s12974-019-1460-1 PMC6449915 30953513

[205] Elabi OF, Karampatsi D, Vercalsteren E, Lietzau G, Nyström T, Klein T, et al. (2023). DPP-4 Inhibitor and Sulfonylurea Differentially Reverse Type 2 Diabetes-Induced Blood-Brain Barrier Leakage and Normalize Capillary Pericyte Coverage. Diabetes, 72:405-414. 10.2337/db22-0674 PMC9935496 36448982

[206] Tian S, Jiang J, Wang J, Zhang Z, Miao Y, Ji X, et al. (2023). Comparison on cognitive outcomes of antidiabetic agents for type 2 diabetes: A systematic review and network meta-analysis. Diabetes Metab Res Rev, 39:e3673. 10.1002/dmrr.3673 37302139

[207] Adamou A, Barkas F, Milionis H, Ntaios G (2024). Glucagon-like peptide-1 receptor agonists and stroke: A systematic review and meta-analysis of cardiovascular outcome trials. Int J Stroke:17474930241253988. 10.1177/17474930241253988 38676552

[208] Stefanou MI, Theodorou A, Malhotra K, Aguiar de Sousa D, Katan M, Palaiodimou L, et al. (2024). Risk of major adverse cardiovascular events and stroke associated with treatment with GLP-1 or the dual GIP/GLP-1 receptor agonist tirzepatide for type 2 diabetes: A systematic review and meta-analysis. Eur Stroke J:23969873241234238. 10.1177/23969873241234238 PMC11418422 38400569

[209] Chen X, Zhao P, Wang W, Guo L, Pan Q (2024). The Antidepressant Effects of GLP-1 Receptor Agonists: A Systematic Review and Meta-Analysis. Am J Geriatr Psychiatry, 32:117-127. 10.1016/j.jagp.2023.08.010 37684186

[210] Yuan Y, Zhang Y, Lei M, Guo X, Yang X, Ouyang C, et al. (2024). Effects of DPP4 Inhibitors as Neuroprotective Drug on Cognitive Impairment in Patients with Type 2 Diabetes Mellitus: A Meta-Analysis and Systematic Review. Int J Endocrinol, 2024:9294113. 10.1155/2024/9294113 PMC10878760 38379936

[211] Li J, Ji C, Zhang W, Lan L, Ge W (2023). Effect of new glucose-lowering drugs on stroke in patients with type 2 diabetes: A systematic review and Meta-analysis. J Diabetes Complications, 37:108362. 10.1016/j.jdiacomp.2022.108362 36462459

[212] Fei Y, Tsoi MF, Cheung BMY (2019). Cardiovascular outcomes in trials of new antidiabetic drug classes: a network meta-analysis. Cardiovasc Diabetol, 18:112. 10.1186/s12933-019-0916-z PMC6714383 31462224

[213] Wium-Andersen IK, Osler M, Jørgensen MB, Rungby J, Wium-Andersen MK (2022). Diabetes, antidiabetic medications and risk of depression - A population-based cohort and nested case-control study. Psychoneuroendocrinology, 140:105715. 10.1016/j.psyneuen.2022.105715 35338947

[214] Mui JV, Li L, Chou OHI, Azfar N, Lee A, Hui J, et al. (2023). Comparing sodium-glucose cotransporter 2 inhibitors and dipeptidyl peptidase-4 inhibitors on new-onset depression: a propensity score-matched study in Hong Kong. Acta Diabetol, 60:917-927. 10.1007/s00592-023-02063-6 PMC10198893 37000300

[215] Shrestha AB, Halder A, Rajak K, Jha SK, Lamichhane R, Oishee AN, et al. (2024). Cardioprotective effects of sodium glucose cotransporter 2 inhibitor versus dipeptidyl peptidase 4 inhibitor in type 2 diabetes: A meta-analysis of comparative safety and efficacy. SAGE Open Med, 12:20503121241261204. 10.1177/20503121241261204 PMC11282519 39070014

[216] Yu AS, Hirayama BA, Timbol G, Liu J, Basarah E, Kepe V, et al. (2010). Functional expression of SGLTs in rat brain. Am J Physiol Cell Physiol, 299:C1277-1284. 10.1152/ajpcell.00296.2010 PMC3006325 20826762

[217] Lin B, Koibuchi N, Hasegawa Y, Sueta D, Toyama K, Uekawa K, et al. (2014). Glycemic control with empagliflozin, a novel selective SGLT2 inhibitor, ameliorates cardiovascular injury and cognitive dysfunction in obese and type 2 diabetic mice. Cardiovasc Diabetol, 13:148. 10.1186/s12933-014-0148-1 PMC4219031 25344694

[218] Nguyen T, Wen S, Gong M, Yuan X, Xu D, Wang C, et al. (2020). Dapagliflozin Activates Neurons in the Central Nervous System and Regulates Cardiovascular Activity by Inhibiting SGLT-2 in Mice. Diabetes Metab Syndr Obes, 13:2781-2799. 10.2147/DMSO.S258593 PMC7425107 32848437

[219] Liu Z, Hua W, Jin S, Wang Y, Pang Y, Wang B, et al. (2024). Canagliflozin protects against hyperglycemia-induced cerebrovascular injury by preventing blood-brain barrier (BBB) disruption via AMPK/Sp1/adenosine A2A receptor. Eur J Pharmacol, 968:176381. 10.1016/j.ejphar.2024.176381 38341077

[220] da Rocha GHO, Loiola RA, de Paula-Silva M, Shimizu F, Kanda T, Vieira A, et al. (2022). Pioglitazone Attenuates the Effects of Peripheral Inflammation in a Human In Vitro Blood-Brain Barrier Model. Int J Mol Sci, 23. 10.3390/ijms232112781 PMC9656730 36361571

[221] Moon JH, Kim HJ, Yang AH, Kim HM, Lee BW, Kang ES, et al. (2012). The effect of rosiglitazone on LRP1 expression and amyloid β uptake in human brain microvascular endothelial cells: a possible role of a low-dose thiazolidinedione for dementia treatment. Int J Neuropsychopharmacol, 15:135-142. 10.1017/S1461145711001611 22040807

[222] Wang H, Chen F, Zhong KL, Tang SS, Hu M, Long Y, et al. (2016). PPARγ agonists regulate bidirectional transport of amyloid-β across the blood-brain barrier and hippocampus plasticity in db/db mice. Br J Pharmacol, 173:372-385. 10.1111/bph.13378 PMC5341223 26507867

[223] Kuate Defo A, Bakula V, Pisaturo A, Labos C, Wing SS, Daskalopoulou SS (2024). Diabetes, antidiabetic medications and risk of dementia: A systematic umbrella review and meta-analysis. Diabetes Obes Metab, 26:441-462. 10.1111/dom.15331 37869901

[224] Lee M, Saver JL, Liao HW, Lin CH, Ovbiagele B (2017). Pioglitazone for Secondary Stroke Prevention: A Systematic Review and Meta-Analysis. Stroke, 48:388-393. 10.1161/STROKEAHA.116.013977 27999139

[225] Bain S, Druyts E, Balijepalli C, Baxter CA, Currie CJ, Das R, et al. (2017). Cardiovascular events and all-cause mortality associated with sulphonylureas compared with other antihyperglycaemic drugs: A Bayesian meta-analysis of survival data. Diabetes Obes Metab, 19:329-335. 10.1111/dom.12821 27862902

[226] Varvaki Rados D, Catani Pinto L, Reck Remonti L, Bauermann Leitão C, Gross JL (2016). The Association between Sulfonylurea Use and All-Cause and Cardiovascular Mortality: A Meta-Analysis with Trial Sequential Analysis of Randomized Clinical Trials. PLoS Med, 13:e1001992. 10.1371/journal.pmed.1001992 PMC4829174 27071029

[227] Catapano JS, Koester SW, Bond KM, Srinivasan VM, Farhadi DS, Rumalla K, et al. (2023). Outcomes in Patients with Aneurysmal Subarachnoid Hemorrhage Receiving Sulfonylureas: A Propensity-Adjusted Analysis. World Neurosurg, 176:e400-e407. 10.1016/j.wneu.2023.05.073 PMC11578856 37236313

[228] Rosado AF, Rosa PB, Platt N, Pierone BC, Neis VB, Severo Rodrigues AL, et al. (2021). Glibenclamide treatment prevents depressive-like behavior and memory impairment induced by chronic unpredictable stress in female mice. Behav Pharmacol, 32:170-181. 10.1097/FBP.0000000000000599 33079735

[229] Xu F, Shen G, Su Z, He Z, Yuan L (2019). Glibenclamide ameliorates the disrupted blood-brain barrier in experimental intracerebral hemorrhage by inhibiting the activation of NLRP3 inflammasome. Brain Behav, 9:e01254. 10.1002/brb3.1254 PMC6456786 30859754

[230] Kuai Z, Xu Y, Zhao Q, Liu J, Guan S, Qiao Y, et al. (2018). Effects of insulin on transcriptional response and permeability in an in vitro model of human blood-brain barrier. J Cell Biochem, 119:5657-5664. 10.1002/jcb.26744 29384214

[231] Sun YN, Liu LB, Xue YX, Wang P (2015). Effects of insulin combined with idebenone on blood-brain barrier permeability in diabetic rats. J Neurosci Res, 93:666-677. 10.1002/jnr.23511 25421718

[232] Liu H, Liu X, Jia L, Liu Y, Yang H, Wang G, et al. (2008). Insulin therapy restores impaired function and expression of P-glycoprotein in blood-brain barrier of experimental diabetes. Biochem Pharmacol, 75:1649-1658. 10.1016/j.bcp.2008.01.004 18299117

[233] Wu S, Stogios N, Hahn M, Navagnanavel J, Emami Z, Chintoh A, et al. (2023). Outcomes and clinical implications of intranasal insulin on cognition in humans: A systematic review and meta-analysis. PLoS One, 18:e0286887. 10.1371/journal.pone.0286887 PMC10306194 37379265

[234] Steineck I, Cederholm J, Eliasson B, Rawshani A, Eeg-Olofsson K, Svensson AM, et al. (2015). Insulin pump therapy, multiple daily injections, and cardiovascular mortality in 18,168 people with type 1 diabetes: observational study. Bmj, 350:h3234. 10.1136/bmj.h3234 PMC4476263 26100640

[235] Liang W, Lo SHS, Tola YO, Chow KM (2021). The effectiveness of self-management programmes for people with type 2 diabetes receiving insulin injection: A systematic review and meta-analysis. Int J Clin Pract, 75:e14636. 10.1111/ijcp.14636 34309961

[236] (2023). Blood pressure targets for prevention of cognitive decline in patients with diabetes and hypertension: Design of the Blood Pressure Control Target in Diabetes (BPROAD) Cognitive Study. J Diabetes, 15:1041-1047. 10.1111/1753-0407.13412 PMC10755604 37737064

[237] Liu J, Li Y, Ge J, Yan X, Zhang H, Zheng X, et al. (2024). Lowering systolic blood pressure to less than 120 mm Hg versus less than 140 mm Hg in patients with high cardiovascular risk with and without diabetes or previous stroke: an open-label, blinded-outcome, randomised trial. Lancet, 404:245-255. 10.1016/S0140-6736(24)01028-6 38945140

[238] Min LJ, Mogi M, Shudou M, Jing F, Tsukuda K, Ohshima K, et al. (2012). Peroxisome proliferator-activated receptor-γ activation with angiotensin II type 1 receptor blockade is pivotal for the prevention of blood-brain barrier impairment and cognitive decline in type 2 diabetic mice. Hypertension, 59:1079-1088. 10.1161/HYPERTENSIONAHA.112.192401 22454480

[239] Awad AS (2006). Role of AT1 receptors in permeability of the blood-brain barrier in diabetic hypertensive rats. Vascul Pharmacol, 45:141-147. 10.1016/j.vph.2006.04.004 16959546

[240] Kayano R, Morofuji Y, Nakagawa S, Fukuda S, Watanabe D, Ozawa H, et al. (2018). In vitro analysis of drugs that improve hyperglycemia-induced blood-brain barrier dysfunction. Biochem Biophys Res Commun, 503:1885-1890. 10.1016/j.bbrc.2018.07.131 30060956

[241] Jain S, Sharma BM, Sharma B (2016). Calcium Channel Blockade and Peroxisome Proliferator Activated Receptor γ Agonism Diminish Cognitive Loss and Preserve Endothelial Function During Diabetes Mellitus. Curr Neurovasc Res, 13:33-44. 10.2174/1567202613666151203233500 26648342

[242] Mooradian AD, Haas MJ, Batejko O, Hovsepyan M, Feman SS (2005). Statins ameliorate endothelial barrier permeability changes in the cerebral tissue of streptozotocin-induced diabetic rats. Diabetes, 54:2977-2982. 10.2337/diabetes.54.10.2977 16186401

[243] Muneeb M, Mansou SM, Saleh S, Mohammed RA (2022). Vitamin D and rosuvastatin alleviate type-II diabetes-induced cognitive dysfunction by modulating neuroinflammation and canonical/noncanonical Wnt/β-catenin signaling. PLoS One, 17:e0277457. 10.1371/journal.pone.0277457 PMC9662739 36374861

[244] Pal K, Mukadam N, Petersen I, Cooper C (2018). Mild cognitive impairment and progression to dementia in people with diabetes, prediabetes and metabolic syndrome: a systematic review and meta-analysis. Soc Psychiatry Psychiatr Epidemiol, 53:1149-1160. 10.1007/s00127-018-1581-3 PMC6208946 30182156

[245] Soroush N, Nekouei Shahraki M, Mohammadi Jouabadi S, Amiri M, Aribas E, Stricker BH, et al. (2024). Statin therapy and cardiovascular protection in type 2 diabetes: The role of baseline LDL-Cholesterol levels. A systematic review and meta-analysis of observational studies. Nutr Metab Cardiovasc Dis. 10.1016/j.numecd.2024.04.015 38866619

[246] Macedo AF, Taylor FC, Casas JP, Adler A, Prieto-Merino D, Ebrahim S (2014). Unintended effects of statins from observational studies in the general population: systematic review and meta-analysis. BMC Med, 12:51. 10.1186/1741-7015-12-51 PMC3998050 24655568

[247] Li W, Qiu J, Li XL, Aday S, Zhang J, Conley G, et al. (2021). BBB pathophysiology-independent delivery of siRNA in traumatic brain injury. Sci Adv, 7. 10.1126/sciadv.abd6889 PMC7775748 33523853

[248] Luan S, Cheng W, Wang C, Gong J, Zhou J (2022). Impact of glucagon-like peptide 1 analogs on cognitive function among patients with type 2 diabetes mellitus: A systematic review and meta-analysis. Front Endocrinol (Lausanne), 13:1047883. 10.3389/fendo.2022.1047883 PMC9650490 36387915

[249] Gonzalez JS, Bebu I, Krause-Steinrauf H, Hoogendoorn CJ, Crespo-Ramos G, Presley C, et al. (2024). Differential Effects of Type 2 Diabetes Treatment Regimens on Diabetes Distress and Depressive Symptoms in the Glycemia Reduction Approaches in Diabetes: A Comparative Effectiveness Study (GRADE). Diabetes Care, 47:610-619. 10.2337/dc23-2459 PMC10973899 38416773

[250] Liu CH, Sung PS, Li YR, Huang WK, Lee TW, Huang CC, et al. (2021). Telmisartan use and risk of dementia in type 2 diabetes patients with hypertension: A population-based cohort study. PLoS Med, 18:e1003707. 10.1371/journal.pmed.1003707 PMC8289120 34280191

[251] Antoniou T, Camacho X, Yao Z, Gomes T, Juurlink DN, Mamdani MM (2013). Comparative effectiveness of angiotensin-receptor blockers for preventing macrovascular disease in patients with diabetes: a population-based cohort study. Cmaj, 185:1035-1041. 10.1503/cmaj.121771 PMC3761008 23836857

[252] Nong J, Glassman PM, Shuvaev VV, Reyes-Esteves S, Descamps HC, Kiseleva RY, et al. (2024). Targeting lipid nanoparticles to the blood-brain barrier to ameliorate acute ischemic stroke. Mol Ther, 32:1344-1358. 10.1016/j.ymthe.2024.03.004 PMC11081939 38454606

[253] Marcos-Contreras OA, Greineder CF, Kiseleva RY, Parhiz H, Walsh LR, Zuluaga-Ramirez V, et al. (2020). Selective targeting of nanomedicine to inflamed cerebral vasculature to enhance the blood-brain barrier. Proc Natl Acad Sci U S A, 117:3405-3414. 10.1073/pnas.1912012117 PMC7035611 32005712

[254] Shen J, Zhao Z, Shang W, Liu C, Zhang B, Zhao L, et al. (2017). Ginsenoside Rg1 nanoparticle penetrating the blood-brain barrier to improve the cerebral function of diabetic rats complicated with cerebral infarction. Int J Nanomedicine, 12:6477-6486. 10.2147/IJN.S139602 PMC5592953 28919749

[255] Ebrahimpour S, Esmaeili A, Beheshti S (2018). Effect of quercetin-conjugated superparamagnetic iron oxide nanoparticles on diabetes-induced learning and memory impairment in rats. Int J Nanomedicine, 13:6311-6324. 10.2147/IJN.S177871 PMC6188001 30349252

[256] Arcambal A, Taïlé J, Couret D, Planesse C, Veeren B, Diotel N, et al. (2020). Protective Effects of Antioxidant Polyphenols against Hyperglycemia-Mediated Alterations in Cerebral Endothelial Cells and a Mouse Stroke Model. Mol Nutr Food Res, 64:e1900779. 10.1002/mnfr.201900779 32447828

[257] VanGilder RL, Kelly KA, Chua MD, Ptachcinski RL, Huber JD (2009). Administration of sesamol improved blood-brain barrier function in streptozotocin-induced diabetic rats. Exp Brain Res, 197:23-34. 10.1007/s00221-009-1866-6 19565232

[258] Rom S, Zuluaga-Ramirez V, Reichenbach NL, Erickson MA, Winfield M, Gajghate S, et al. (2018). Secoisolariciresinol diglucoside is a blood-brain barrier protective and anti-inflammatory agent: implications for neuroinflammation. J Neuroinflammation, 15:25. 10.1186/s12974-018-1065-0 PMC5787274 29373982

[259] Shaheryar ZA, Khan MA, Hameed H, Mushtaq MN, Muhammad S, Shazly GA, et al. (2023). Natural Fatty Acid Guards against Brain Endothelial Cell Death and Microvascular Pathology following Ischemic Insult in the Presence of Acute Hyperglycemia. Biomedicines, 11. 10.3390/biomedicines11123342 PMC10742291 38137563

[260] Wang H, Chen F, Du YF, Long Y, Reed MN, Hu M, et al. (2018). Targeted inhibition of RAGE reduces amyloid-β influx across the blood-brain barrier and improves cognitive deficits in db/db mice. Neuropharmacology, 131:143-153. 10.1016/j.neuropharm.2017.12.026 29248482

[261] Ward R, Li W, Abdul Y, Jackson L, Dong G, Jamil S, et al. (2019). NLRP3 inflammasome inhibition with MCC950 improves diabetes-mediated cognitive impairment and vasoneuronal remodeling after ischemia. Pharmacol Res, 142:237-250. 10.1016/j.phrs.2019.01.035 PMC6486792 30818045

[262] Zhao Q, Zhang F, Yu Z, Guo S, Liu N, Jiang Y, et al. (2019). HDAC3 inhibition prevents blood-brain barrier permeability through Nrf2 activation in type 2 diabetes male mice. J Neuroinflammation, 16:103. 10.1186/s12974-019-1495-3 PMC6525453 31101061

[263] Zhao Z, Huang G, Wang B, Zhong Y (2013). Inhibition of NF-kappaB activation by Pyrrolidine dithiocarbamate partially attenuates hippocampal MMP-9 activation and improves cognitive deficits in streptozotocin-induced diabetic rats. Behav Brain Res, 238:44-47. 10.1016/j.bbr.2012.10.018 23089644

[264] Zheng H, Xu P, Jiang Q, Xu Q, Zheng Y, Yan J, et al. (2021). Depletion of acetate-producing bacteria from the gut microbiota facilitates cognitive impairment through the gut-brain neural mechanism in diabetic mice. Microbiome, 9:145. 10.1186/s40168-021-01088-9 PMC8235853 34172092

[265] de Cossío LF, Fourrier C, Sauvant J, Everard A, Capuron L, Cani PD, et al. (2017). Impact of prebiotics on metabolic and behavioral alterations in a mouse model of metabolic syndrome. Brain, Behavior, and Immunity, 64:33-49. 10.1016/j.bbi.2016.12.022 28027925

[266] Morshedi M, Valenlia KB, Hosseinifard ES, Shahabi P, Abbasi MM, Ghorbani M, et al. (2018). Beneficial psychological effects of novel psychobiotics in diabetic rats: the interaction among the gut, blood and amygdala. J Nutr Biochem, 57:145-152. 10.1016/j.jnutbio.2018.03.022 29730508

[267] Hosseinifard ES, Morshedi M, Bavafa-Valenlia K, Saghafi-Asl M (2019). The novel insight into anti-inflammatory and anxiolytic effects of psychobiotics in diabetic rats: possible link between gut microbiota and brain regions. Eur J Nutr, 58:3361-3375. 10.1007/s00394-019-01924-7 30826905

[268] Wątroba M, Grabowska AD, Szukiewicz D (2023). Effects of Diabetes Mellitus-Related Dysglycemia on the Functions of Blood-Brain Barrier and the Risk of Dementia. Int J Mol Sci, 24. 10.3390/ijms241210069 PMC10299009 37373216

